# Management of de Quervain Tenosynovitis: A Systematic Review and Network Meta-Analysis

**DOI:** 10.1001/jamanetworkopen.2023.37001

**Published:** 2023-10-27

**Authors:** Dimitris Challoumas, Rohan Ramasubbu, Elliot Rooney, Emily Seymour-Jackson, Amit Putti, Neal L. Millar

**Affiliations:** 1 School of Infection and Immunity, College of Medicine, Veterinary and Life Sciences, University of Glasgow, Glasgow, Scotland; 2 Department of Orthopaedic Surgery, Forth Valley Royal Hospital, Larbert, Scotland

## Abstract

**Importance:**

There is a plethora of treatment options for patients with de Quervain tenosynovitis (DQT), but there are limited data on their effectiveness and no definitive management guidelines.

**Objective:**

To assess and compare the effectiveness associated with available treatment options for DQT to guide musculoskeletal practitioners and inform guidelines.

**Data Sources:**

Medline, Embase, PubMed, Cochrane Central, Scopus, OpenGrey.eu, and WorldCat.org were searched for published studies, and the World Health Organization International Clinical Trials Registry Platform, ClinicalTrials.gov, The European Union Clinical Trials Register, and the ISRCTN registry were searched for unpublished and ongoing studies from inception to August 2022.

**Study Selection:**

All randomized clinical trials assessing the effectiveness of any intervention for the management of DQT.

**Data Extraction and Synthesis:**

This study was prospectively registered on PROSPERO and conducted and reported per Preferred Reporting Items for Systematic Reviews and Meta-Analyses Extension Statement for Reporting of Systematic Reviews Incorporating Network Meta-analyses of Health Care Interventions (PRISMA-NMA) and PRISMA in Exercise, Rehabilitation, Sport Medicine and Sports Science (PERSIST) guidance. The Cochrane Risk of Bias tool and the Grading of Recommendations, Assessment, Development, and Evaluations tool were used for risk of bias and certainty of evidence assessment for each outcome.

**Main Outcomes and Measures:**

Pairwise and network meta-analyses were performed for patient-reported pain using a visual analogue scale (VAS) and for function using the quick disabilities of the arm, shoulder, and hand (Q-DASH) scale. Mean differences (MD) with their 95% CIs were calculated for the pairwise meta-analyses.

**Results:**

A total of 30 studies with 1663 patients (mean [SD] age, 46 [7] years; 80% female) were included, of which 19 studies were included in quantitative analyses. From the pairwise meta-analyses, based on evidence of moderate certainty, adding thumb spica immobilization for 3 to 4 weeks to a corticosteroid injection (CSI) was associated with statistically but not clinically significant functional benefits in the short-term (MD, 10.5 [95% CI, 6.8-14.1] points) and mid-term (MD, 9.4 [95% CI, 7.0-11.9] points). In the network meta-analysis, interventions that included ultrasonography-guided CSI ranked at the top for pain. CSI with thumb spica immobilization had the highest probability of being the most effective intervention for short- and mid-term function.

**Conclusions and Relevance:**

This network meta-analysis found that adding a short period of thumb spica immobilization to CSI was associated with statistically but not clinically significant short- and mid-term benefits. These findings suggest that administration of CSI followed by 3 to 4 weeks immobilization should be considered as a first-line treatment for patients with DQT.

## Key Points

**Question:**

What treatment modalities for de Quervain tenosynovitis are associated with better outcomes compared with other treatments?

**Findings:**

This systematic review and network meta-analysis of 30 studies with 1663 patients found that adding thumb spica immobilization to a local corticosteroid injection was associated with significant pain-relieving and functional benefits. Administering the corticosteroid injection using ultrasonographic guidance was associated with greater pain reduction than conventional injections.

**Meaning:**

These findings suggest that patients with de Quervain tenosynovitis should receive a local corticosteroid injection with thumb spica immobilization for 3 to 4 weeks as first-line treatment.

## Teaser

This systematic review and meta-analysis assesses and compares the effectiveness associated with available treatment options for de Quervain tenosynovitis.

## Introduction

De Quervain tenosynovitis (DQT) is a stenosing overuse condition of the synovial sheath of the first extensor compartment of the wrist affecting the extensor pollicis brevis (EPB) and abductor pollicis longus tendons.^1^ The exact pathophysiology of DQT is unknown, but it appears to be related to thickening of the tendon sheath and the overlying extensor retinaculum, as well as thinning and degeneration of the affected tendons.^2^ Involvement of inflammation remains controversial but intrinsic degeneration due to overuse appears to be the most likely mechanism.^3^ Possible associations have been made between a separate, septated EPB subcompartment and DQT; however, increasing evidence suggests that this is probably a normal anatomical variant.^4^ DQT manifests with pain and tenderness over and proximal to the radial styloid, and while its diagnosis is predominantly clinical, imaging modalities, such as ultrasonography and magnetic resonance imaging, can be useful where there is diagnostic uncertainty.^1^

Definitive guidelines for the management of DQT do not exist. In the published consensus statement from the European HANDGUIDE study,^1^ all experts who participated agreed that all patients with DQT should be given instructions about activity, function, and pain and these should be accompanied by 1 or more of the following interventions: nonsteroidal anti-inflammatory drugs (NSAIDs), splinting, corticosteroid injection (CSI), and surgery.^1^ The intervention should be chosen based on severity, duration of DQT, and previous treatments given. Consensus was reached on a therapeutic hierarchy, which starts with instructions plus NSAIDs and finishes with surgery.^1^

An increasing number of treatment options for DQT is becoming available, such as hyaluronic acid injections, extracorporeal shockwave therapy, acupuncture, ultrasonographic therapy, and laser therapy, all with limited evidence on their effectiveness. Therefore, management decisions can be challenging, given the several available treatment modalities and their possible combinations. Our aim was to present the highest quality of evidence on the comparative effectiveness associated with available interventions for DQT to facilitate clinical practice decisions and contribute to future guidelines.

## Methods

This systematic review and network meta-analysis was registered on PROSPERO (registration No. CRD42022346986). This study is reported following the Preferred Reporting Items for Systematic Reviews and Meta-Analyses Extension Statement for Reporting of Systematic Reviews Incorporating Network Meta-analyses of Health Care Interventions (PRISMA-NMA) and PRISMA in Exercise, Rehabilitation, Sport Medicine and Sports Science (PERSIST) reporting guidelines. Outcomes of interest were patient-reported pain, assessed using a visual analogue scale (VAS; range, 0-10; higher score indicates worse pain),^5^ and function, assessed using the quick disabilities of the arm, shoulder, and hand (Q-DASH) scale (range, 0-80; higher score indicates worse function).^6^

**Figure 1.**
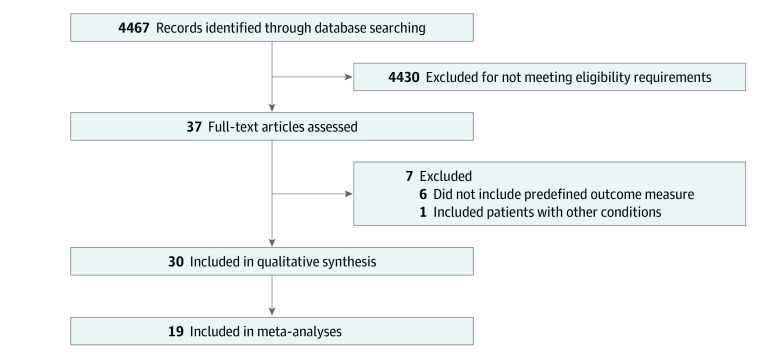
Flowchart of Study Selection Process

We searched Medline, Embase, PubMed, Cochrane Central, Scopus, OpenGrey.eu, and WorldCat.org for published studies, and we searched the WHO International Clinical Trials Registry Platform, ClinicalTrials.gov, European Union Clinical Trials Register, and ISRCTN registry for unpublished and ongoing studies from inception to August 2022 (Figure 1). The Cochrane Risk of Bias (ROB) tool^7^ and the Grading of Recommendations, Assessment, Development, and Evaluations (GRADE) tool^8^ were used for risk of bias and certainty of evidence assessment for each outcome. Complete details on eligibility criteria, the literature search, data extraction, data handling, protocol deviations, and risk of bias and strength of evidence assessments are provided in the eMethods in Supplement 1.

### Statistical Analysis

The Review Manager software version 5 (RevMan) was used to calculate pooled mean differences (MDs) with 95% CIs and generate forest plots for pairwise meta-analyses and their accompanying heterogeneity tests (χ^2^ and *I*^2^) and *P* values. Stata software version 16.1 (StataCorp) with multivariate random-effects meta-regression was used for network meta-analyses. Statistical significance was set at 2-sided *P* < .05. Data were analyzed from August 2022 to June 2023.

## Results

A total of 37 studies^9,10,11,12,13,14,15,16,17,18,19,20,21,22,23,24,25,26,27,28,29,30,31,32,33,34,35,36,37,38,39,40,41,42,43,44,45^ were initially found to be eligible. Of those, 7 studies^9,10,11,12,13,14,15^ did not include 1 of our predefined outcome measures or included patients with conditions other than DQT and did not analyze data separately and were therefore excluded (Figure 1). A total of 30 studies^17,18,19,20,21,22,23,24,25,26,27,28,29,30,31,32,33,34,35,36,37,38,39,40,41,42,43,44,45,46^ with 1663 patients (mean age [SD] age, 46 [7] years; 80% female) were included in further data collection. eTable 1 in Supplement 1 summarizes the patient, intervention, comparator, and outcome characteristics and the individual findings of these studies for pain and function, as well as the results of our pairwise meta-analyses where pooling of studies was possible. There was a total of 25 treatment comparisons. Study publication year ranged from 2009 to 2022. eTable 2 in Supplement 1 shows the risk of bias assessment results of the ROB assessment. Of 19 studies^16,17,18,19,21,24,25,26,27,29,35,36,37,38,39,41,42,43,45^ that participated in quantitative analyses (pairwise or network meta-analyses), 1 study was of low ROB and 8 studies were of high overall ROB, while the remaining 10 studies were rated as having some concerns (eTable 2 in Supplement 1).

### Pairwise Meta-Analyses

**Figure 2.**
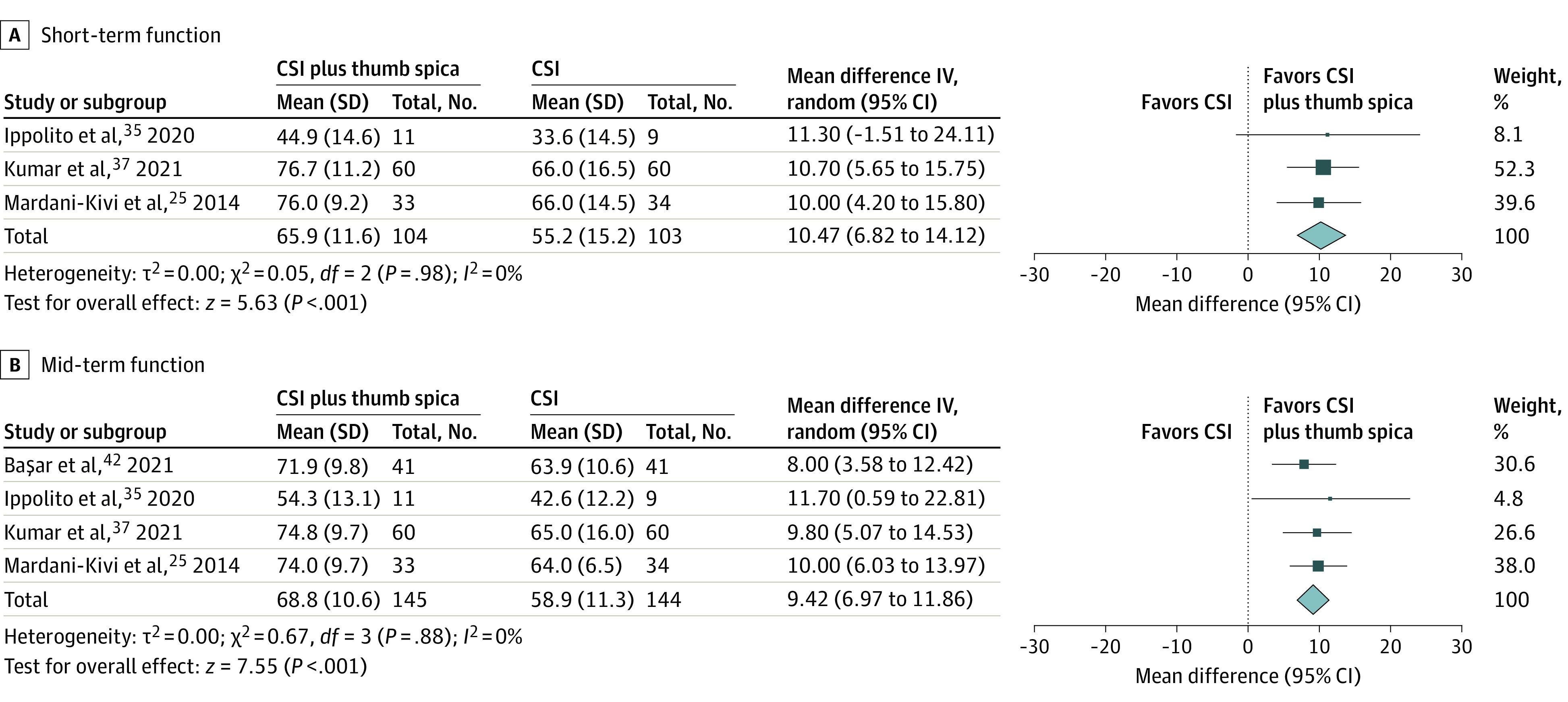
Results of Pairwise Meta-Analysis of Function Outcomes of Corticosteroid Injection (CSI) Plus Thumb Spica Immobilization vs CSI Alone Function outcomes were assessed using the quick disabilities of the arm, shoulder, and hand scale (range, 0-80; higher score indicates worse function).^6^ Short-term outcomes were defined as 0 to 12 weeks; mid-term, 13 weeks to 12 months. Each square represents the point estimate of the result of that study; size of square is proportional to the contribution of the study to the overall result, dependent on the population size and SD. Whiskers indicate 95% CIs. The diamond represents the combined point estimate and 95% CI.

Figure 2 illustrates the results of the pairwise meta-analyses of comparisons that were based on either moderate or high certainty of evidence, with their forest plots and accompanying statistical heterogeneity tests. eFigures 2 through 7 in Supplement 1 show the forest plots of the pairwise meta-analyses of comparisons that were based on low or very low certainty of evidence. eTable 3 in Supplement 1 summarizes the certainty of evidence assessment process for all pairwise meta-analyses. Information on complications is provided in the eAppendix in Supplement 1.

#### CSI Plus Thumb Spica Immobilization vs CSI Alone

Among 4 studies^25,35,37,42^ in this meta-analysis; 1 was of high overall ROB and 3 were of some concerns of ROB. Postinjection immobilization was for either 3 weeks^25,35^ or 4 weeks,^37,42^ in a cast,^25,37^ a splint,^42^ or either.^35^ For both short-term and mid-term pain, the group that included immobilization was associated with statistically but not clinically significant improvements in VAS scores (short-term MD, 1.3 [95% CI, 0.4-2.1] points; *I*^2^ = 86%; 3 studies^25,35,37^; 207 participants; very low certainty of evidence; mid-term MD, 1.2 [95% CI, 0.3-2.2] points; *I*^2^ = 93%; 4 studies^25,35,37,42^; 289 participants; very low certainty of evidence). The benefits of adding immobilization to CSI were also evident for short-term and mid-term function based on Q-DASH scores, and this was also statistically but not clinically significant (short-term MD, 10.5 [95% CI, 6.8-14.1] points; *I*^2^ = 0%; 3 studies^25,35,37^; 207 participants; moderate certainty of evidence; mid-term MD, 9.4 [95% CI, 7.0, 11.9] points; I^2^ = 0%; 4 studies^25,35,37,42^; 289 participants; moderate certainty of evidence).

#### Ultrasonography-Guided CSI vs Conventional CSI

Two studies^21,38^ with some concerns in their overall ROB assessments were pooled for the comparison of ultrasonography-guided vs conventional CSI. For short-term pain VAS scores, although the pooled outcome favored the ultrasonography-guided group at clinical significance, this was not statistically significant due to a very wide CI (MD, 2.1 [95% CI, −0.5 to 4.6] points; *I*^2^ = 91%; 2 studies^21,38^; 92 participants; very low certainty of evidence).

#### Open Surgery With Transverse Incision vs Longitudinal Incision

Two studies^19,26^ of overall high ROB were pooled for the comparison of transverse vs longitudinal incision for open surgery. Despite the large ORs for total complications (OR, 6.8 [95% CI, 0.9 to 48.1]; *I*^2^ = 64%; 2 studies^19,26^; very low certainty of evidence) and superficial radial nerve injury (OR, 7.7 [95% CI, 0.9, 64.0]; *I*^2^ = 0%; 2 studies^19,26^; very low certainty of evidence) in favor of longitudinal incisions, these did not reach statistical significance due to very wide CIs. The incidence of hypertrophic scar was similar between incision types (OR 2.0 [95% CI, 0.4-10.8]; *I*^2^ = 47%; 2 studies^19,26^; very low certainty of evidence).

### Network Meta-Analyses

A total of 17 studies^16,17,18,21,24,25,27,29,35,36,37,38,39,41,42,43,45^ were included in network meta-analyses, which were performed separately for short-term pain (15 studies^16,17,18,21,24,25,27,29,35,36,37,38,39,41,43,45^; 14 interventions), mid-term pain (9 studies^17,18,25,27,35,37,41,42,43^; 7 interventions), short-term function (8 studies^24,25,27,35,36,37,41,43^; 7 interventions), and mid-term function (6 studies^25,27,35,37,41,42^; 3 interventions). eFigures 17 through 20 in Supplement 1 show the comparative treatment class effects for short-term pain, mid-term pain, short-term function, and mid-term function. eTables 4 through 7 in Supplement 1 represent the summary of findings of the network meta-analyses, comparing all included interventions with the reference comparator (conventional CSI) for each outcome at each follow up time period.

#### Short-Term Pain

**Figure 3.**
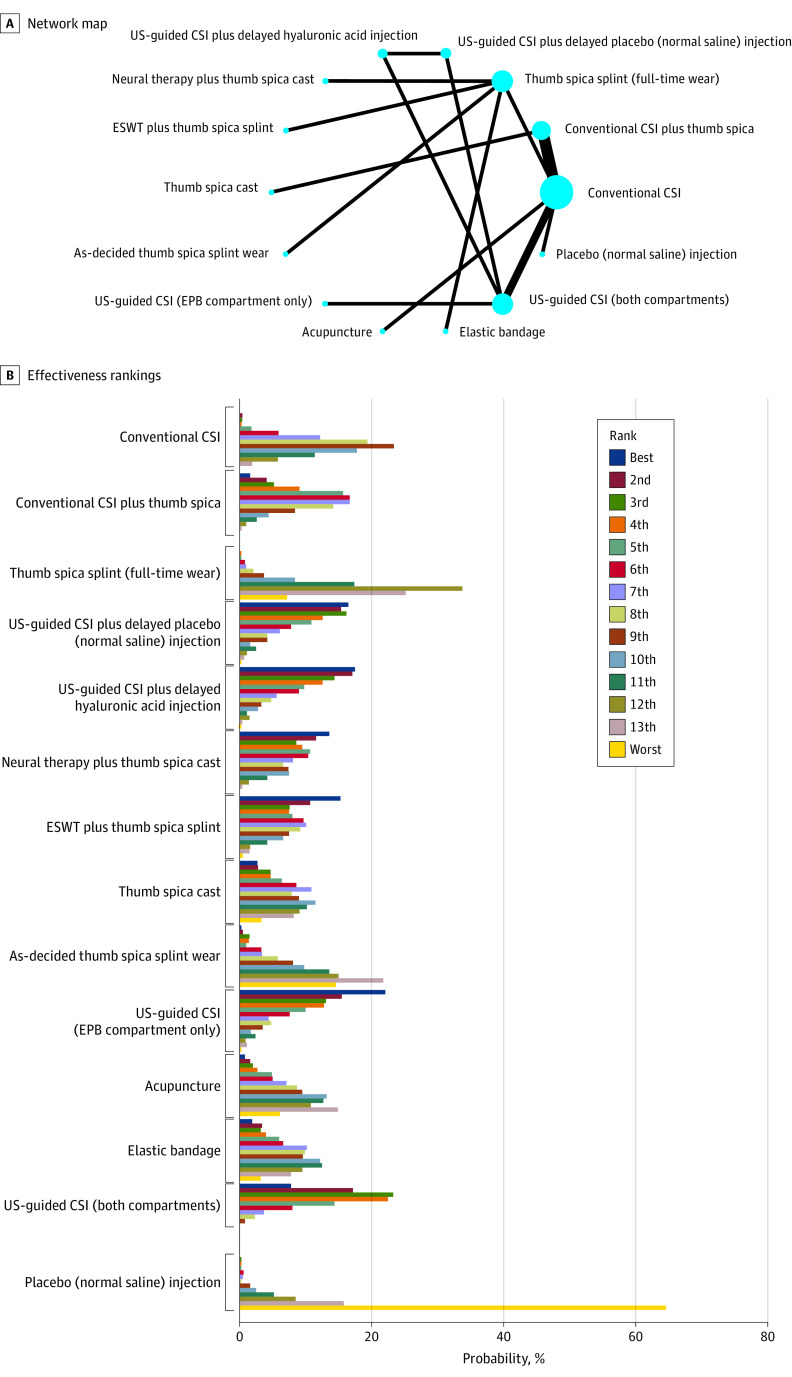
Effectiveness of Interventions for de Quervain Tenosynovitis for Short-Term Pain Short-term was defined as 0 to 12 weeks. A, Diameter of the circle represents the number of studies assessing that intervention; thickness of the line, number of studies assessing that comparison of interventions. CSI indicates conventional corticosteroid injection; EPB, extensor pollicis brevis; ESWT, extracorporeal shockwave therapy; US, ultrasonography.

For pain at 0 to 12 weeks, the most effective interventions were ultrasonography-guided CSI in EPB compartment only (where there is subcompartmentalization), ultrasonography-guided CSI injection plus delayed injection of hyaluronic acid, ultrasonography-guided CSI injection plus delayed injection of normal saline, neural therapy (local anesthetic therapy directed at the autonomic nervous system) plus thumb spica splint, and extracorporeal shockwave therapy plus thumb spica splint. Of these, ultrasonography-guided CSI in EPB compartment only (where there is subcompartmentalization) had the highest probability (22%) of being the most effective. Placebo injection (normal saline) had the highest probability of being the least effective, followed by as-decided thumb spica splint wear and full-time thumb spica splint wear. Figure 3 details the network map, the network forest plots, and the rank bar graphs for short-term pain.

From the combined direct and indirect comparisons neural therapy plus thumb spica splint, extracorporeal shockwave therapy plus thumb spica splint, and ultrasonography-guided CSI were each found to be clinically and statistically significantly superior to thumb spica splint alone. Additionally, ultrasonography-guided CSI was superior to conventional CSI, and conventional CSI with or without thumb spica, ultrasonography-guided CSI with or without delayed injection of normal saline or hyaluronic acid, neural therapy plus thumb spica splint, ultrasonography-guided CSI in EPB compartment only or in both compartments (where there is subcompartmentalization), and ultrasonography-guided CSI were each superior to placebo saline injection alone (eFigure 17 in Supplement 1).

#### Mid-Term Pain

Neural therapy plus thumb spica splint ranked at the top as having the highest probability of being the most effective intervention for pain at 13 to 52 weeks, followed by conventional CSI plus thumb spica immobilization. Thumb spica splint and thumb spica cast had the highest probabilities of being the least effective interventions. eFigures 8 through 10 in Supplement 1 show the network map, the network forest plots, and the rank bar graphs for short-term pain. Out of all combined direct and indirect comparisons, only the comparison between neural therapy plus thumb spica splint vs thumb spica splint alone was statistically and clinically significant, favoring the neural therapy (eFigure 18 in Supplement 1).

#### Short-Term Function

Conventional CSI plus thumb spica immobilization had the highest probability of being the most effective intervention for function at 0 to 12 weeks, followed by acupuncture. Thumb spica splint had the highest probability of being the least effective. eFigures 11 to 13 in Supplement 1 show the network map, the network forest plots, and the rank bar graphs for short-term pain.

From the combined direct and indirect comparisons, conventional CSI plus thumb spica immobilization was clinical and statistically superior to thumb spica cast and thumb spica splint with full-time wear or as-decided wear (eFigure 19 in Supplement 1).

#### Mid-Term Function

Of 3 included interventions, conventional CSI plus thumb spica immobilization had the highest probability of being the most effective intervention and thumb spica splint had the highest probability of being the least effective for function at 13 to 52 weeks. eFigures 14 to 16 in Supplement 1 show the network map, the network forest plots, and the rank bar graphs for short-term pain. There were no combined direct and indirect comparisons that produced both statistically and clinically significant results (eFigure 20 in Supplement 1).

## Discussion

This is systematic review and network meta-analysis on the management of DQT is the largest of its kind to date, to our knowledge. We found through direct comparisons that adding thumb spica immobilization for 3 to 4 weeks to a CSI was associated with benefits for pain and function in the short- and mid-term. While these differences were statistically significant, they did not reach clinical significance due to our predefined minimal clinically important differences for pain and function. Our results were based on low certainty of evidence for pain and moderate certainty of evidence for function; therefore, only function-related recommendations for clinical practice can be considered strong. In the network meta-analysis for short-term pain, interventions that included ultrasonography-guided CSI ranked at the top. Placebo injection (normal saline), and thumb spica immobilization alone (splint or cast) had the highest probability of being the least effective interventions. Data for promising treatments that ranked high in the network meta-analyses, such as neural therapy and extracorporeal shockwave therapy, originated from single studies of high overall ROB; therefore, recommendations for their use cannot be made at this point.

Surgical interventions were not eligible for inclusion in pairwise meta-analyses, since no comparison of the same 2 interventions was assessed by any more than 1 study. Additionally, they could not be included in the network meta-analyses because the studies that assessed surgical interventions did not include any of the other interventions that already participated in our network. The RCT by Kang et al^22^ compared open and endoscopic surgical release for DQT and found better short-term outcomes in the open release group but similar mid-term outcomes. The endoscopic group also had fewer superficial radial nerve complications and greater scar satisfaction.^22^ The RCT by Lu et al^32^ demonstrated possible additional benefits of adding a platelet-rich plasma injection to open surgical release for DQT at mid-term follow-up.^32^ A 2019 study by Kim et al^34^ showed that a dorsoulnar incision of the retinaculum did not have better outcomes compared with a midline incision and neither did postoperative immobilization. A 2016 study by Kumar^26^ compared longitudinal and transverse incisions in patients with DQT and found that the latter had a greater number of total complications, including superficial radial nerve injury, vein injury and scar hypertrophy. Similarly, in a 2011 RCT, Abrisham et al^19^ found that transverse incisions were associated with a greater total number of complications than longitudinal incisions. Finally, an RCT^44^ comparing pulley release vs pulley reconstruction found no difference in clinical outcomes between groups. In a systematic review,^46^ surgical release for DQT has been shown to be highly effective, associated with full resolution of symptoms in up to 95% of patients; however, due to its potential complications, especially injury to the superficial radial nerve, it should be reserved for patients for whom nonsurgical treatment has failed.

The overall incidence of a separate EPB subcompartment within the first extensor compartment has been reported to be as high as 80%, with up to 90% of patients experiencing DQT and 70% of asymptomatic patients having a separate EPB.^47^ This high incidence implies that this should perhaps be regarded as an expectant anatomical component of the normal wrist rather than a variant. An association between this separate subcompartment and EPB acting as a thumb interphalangeal joint extensor has also been reported.^48^ Some researchers have speculated that DQT is secondary to EPB entrapment alone, as evidence suggests that surgically releasing the septated EPB subcompartment only and administering ultrasonography-guided CSI into the EPB subcompartment only are as effective as release of the subcompartment and the first extensor compartment sheath plus CSI into both the subcompartment and the main first extensor compartment.^45,49^ Indeed, CSI into the EPB subcompartment alone ranked among the top interventions in our network meta-analysis for short-term pain.^45^ Finally, a more dorsal incision to the extensor retinaculum has been recommended to avoid tendon subluxation, or even a partial excision of the extensor retinaculum to prevent reannealing of the retinaculum and recurrent symptoms; however, there is currently no convincing evidence to support these recommendations.^34,50^

Although oral NSAIDs have been recommended as first-line treatment for DQT, their use is not supported by the existing literature.^1^ An RCT by Ansari^11^ found that treatment of DQT with NSAIDs, splinting, and local application of ointment had much worse outcomes in terms of treatment success at 1 and 2 weeks compared with a CSI. Another RCT^14^ comparing CSI with or without oral NSAID found no additional benefits associated with the use of the NSAID. Finally, an RCT^51^ that was published after we performed our literature search and was therefore not included in our study found that a CSI was more effective for pain, function, and grip strength at 6 weeks than an NSAID injection.

The findings of previously published systematic reviews of RCTs were largely limited by inadequate evidence. An early Cochrane review^52^ only included 1 RCT of CSI vs thumb spica immobilization in pregnant or postpartum patients, and the superiority of the CSI could not be generalized to the wider population. Similarly, a systematic review^53^ on surgical outcomes for DQT was unable to make any useful conclusions for pain or function since only 3 studies were eligible for inclusion. Another systematic review^54^ of 2 RCTs found that CSI was more effective than splinting. A study by Cavaleri et al^55^ showed that both thumb spica immobilization, acupuncture, and CSI were associated with improved pain and function but CSI plus orthoses interventions were the most effective, which is in agreement with our results. A systematic review^46^ of all study types found that surgery for DQT was effective in up to 95% of patients, and there were no differences in outcomes or complications among different types of surgery and incision. Finally, a study by Huisstede et al^56^ reported moderate evidence for the effectiveness of CSI in DQT in the very short-term, and for adding splinting to CSI in the short- and mid-term.

**Figure 4.**
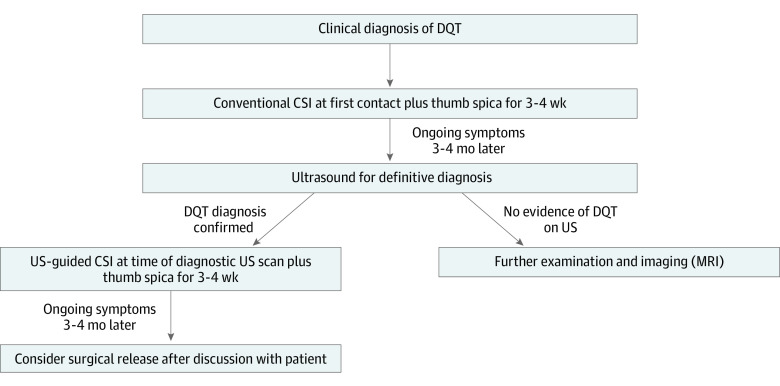
Suggested Treatment Algorithm for de Quervain Tenosynovitis (DQT) CSI indicates conventional corticosteroid; MRI, magnetic resonance imaging; US, ultrasonography.

Based on our findings, we recommend that clinicians offer patients with DQT of any chronicity a conventional CSI at first contact. This should be supplemented with thumb spica immobilization for 3 to 4 weeks in the form of a full-time (minus grooming and simple daily range-of-movement exercises) thumb spica splint that includes the wrist and the thumb metacarpophalangeal joint but not the thumb interphalangeal joint. If the symptoms persist 3 to 4 months later, we recommend that a diagnostic ultrasonographic scan is performed, at which point a further ultrasonography-guided CSI can be administered on confirmation of the diagnosis of DQT. This should also be supplemented with a thumb spica splint for 3 to 4 weeks. If this does not result in resolution of symptoms within 3 to 4 months, then surgical release is recommended. No definitive recommendations can be provided for the type of surgery or type of incision due to inadequate evidence. All patients should be warned about complications of surgery as part of the informed consent process, especially injury to the superficial radial nerve and its consequences. At all stages of treatment, advice about lifestyle modifications should be provided to limit overuse of the affected tendons. Figure 4 illustrates our recommended management pathway.

### Limitations

Our study has some limitations. Chronicity of the condition, which could influence effectiveness of interventions, was not considered and neither was type, number, or dose of CSIs. Similarly, simultaneous consumption of NSAIDs was not controlled for, as the included studies did not provide relevant data. Additionally, not all interventions were available for participation in all networks; specifically surgical interventions could not be included in any quantitative analyses for pain and function. Furthermore, the diagnostic criteria of some studies may have been inadequate, having only included the Finkelstein test. However, we did include all eligible studies that derived from a thorough literature search and combined data appropriately in quantitative analyses that included a network meta-analysis. Additionally, we performed detailed ROB and certainty of evidence assessments for each outcome separately.

## Conclusions

In this systematic review and network meta-analysis of RCTs, we found that adding a short period of thumb spica immobilization to a CSI was associated with significant benefits and administering the CSI with ultrasonographic guidance was associated with superior results compared with conventional CSI. CSI with thumb spica immobilization had the highest probability of being the most effective treatment for function and also ranked very highly for pain relief among treatments in analyses. Therefore, we recommend the use of CSI with thumb spica immobilization for 3 to 4 weeks as first-line treatment for patients with DQT. Surgery should be used only when nonsurgical management fails. Further research should take into account chronicity of the condition, as this may influence effectiveness of specific interventions, and NSAIDs should both be assessed as a management option for DQT, with or without thumb spica immobilization, and controlled for in studies where other interventions are assessed. Finally, more high-quality RCTs should be conducted to investigate the potential benefits of administering CSIs with ultrasonographic guidance and comparing different surgical approaches so that conclusions can be made with higher certainty of evidence.

## Notes

Supplement 1.
eMethods.
**eAppendix.** Complications **eFigure 1.** Network Forest Plots of Effectiveness of Interventions for de Quervain Tenosynovitis for Short-Term Pain **eFigure 2.** Results of Pairwise Meta-Analysis of Short-Term Pain of CSI + Thumb Spica Immobilization vs CSI Alone **eFigure 3.** Results of Pairwise Meta-Analysis of Mid-Term Pain of CSI + Thumb Spica Immobilization vs CSI Alone **eFigure 4.** Results of Pairwise Meta-Analysis of Short-Term Pain of US-Guided CSI vs Conventional CSI Alone **eFigure 5.** Results of Pairwise Meta-Analysis of Mid-Term Total Complications of Transverse vs Longitudinal Skin Incision **eFigure 6.** Results of Pairwise Meta-Analysis of Mid-Term Superficial Radial Nerve Injury of Transverse vs Longitudinal Skin Incision **eFigure 7.** Results of Pairwise Meta-Analysis of Mid-Term Scar Hypertrophy of Transverse vs Longitudinal Skin Incision **eFigure 8.** Network Map of Effectiveness of Interventions for de Quervain Tenosynovitis for Mid-Term Pain **eFigure 9.** Network Forest Plots of Effectiveness of Interventions for de Quervain Tenosynovitis for Mid-Term Pain **eFigure 10.** Rank Bars of Effectiveness of Interventions for de Quervain Tenosynovitis for Mid-Term Pain **eFigure 11.** Network Map of Effectiveness of Interventions for de Quervain Tenosynovitis for Short-Term Function **eFigure 12.** Network Forest Plots of Effectiveness of Interventions for de Quervain Tenosynovitis for Short-Term Function **eFigure 13.** Rank Bars of Effectiveness of Interventions for de Quervain Tenosynovitis for Short-Term Function **eFigure 14.** Network Map of Effectiveness of Interventions for de Quervain Tenosynovitis for Mid-Term Function **eFigure 15.** Network Forest Plots of Effectiveness of Interventions for de Quervain Tenosynovitis for Mid-Term Function **eFigure 16.** Rank Bars of Effectiveness of Interventions for de Quervain Tenosynovitis for Mid-Term Function **eFigure 17.** Comparative Treatment Class Effects for Short-Term Pain **eFigure 18.** Comparative Treatment Class Effects for Mid-Term Pain **eFigure 19.** Comparative Treatment Class Effects for Short-Term Function **eFigure 20.** Comparative Treatment Class Effects for Mid-Term Function **eTable 1.** Results of Each Study Showing the Mean Difference Between the Compared Interventions for Pain and Function **eTable 2.** Results of Risk of Bias Assessment Using the Cochrane ROB 2 Tool **eTable 3.** Results of Certainty of Evidence Assessment Using the GRADE Tool **eTable 4.** Summary of Findings for Short-Term Pain **eTable 5.** Summary of Findings for Mid-Term Pain **eTable 6.** Summary of Findings for Short-Term Function **eTable 7.** Summary of Findings for Mid-Term Function

Supplement 2.
Data Sharing Statement

## References

[1] Huisstede BMA, Coert JH, Fridén J, Hoogvliet P; European HANDGUIDE Group. Consensus on a multidisciplinary treatment guideline for de Quervain disease: results from the European HANDGUIDE study. Phys Ther. 2014;94(8):1095-1110. doi:10.2522/ptj.20130069 24700135

[2] Skef S, Ie K, Sauereisen S, Shelesky G, Haugh A. Treatments for de Quervain tenosynovitis. Am Fam Physician. 2018;97(12). 30216006

[3] Hogrefe C, Jones EM. Tendinopathy and Bursitis. In: Wall RM, Hockberger RS, Gausche-Hill M, . Rosen’s Emergency Medicine: Concepts and Clinical Practice. Elsevier; 2018:1392-1401.

[4] Beutel BG, Doscher ME, Melone CP Jr. Prevalence of a septated first dorsal compartment among patients with and without de Quervain tenosynovitis: an in vivo anatomical study. Hand (N Y). 2020;15(3):348-352. doi:10.1177/1558944718810864 30428712 PMC7225884

[5] Tashjian RZ, Deloach J, Porucznik CA, Powell AP. Minimal clinically important differences (MCID) and patient acceptable symptomatic state (PASS) for visual analog scales (VAS) measuring pain in patients treated for rotator cuff disease. J Shoulder Elbow Surg. 2009;18(6):927-932. doi:10.1016/j.jse.2009.03.021 19535272

[6] Franchignoni F, Vercelli S, Giordano A, Sartorio F, Bravini E, Ferriero G. Minimal clinically important difference of the disabilities of the arm, shoulder and hand outcome measure (DASH) and its shortened version (QuickDASH). J Orthop Sports Phys Ther. 2014;44(1):30-39. doi:10.2519/jospt.2014.4893 24175606

[7] Sterne JAC, Savović J, Page MJ, . ROB 2: a revised tool for assessing risk of bias in randomised trials. BMJ. 2019;366:l4898. doi:10.1136/bmj.l4898 31462531

[8] Puhan MA, Schünemann HJ, Murad MH, ; GRADE Working Group. A GRADE Working Group approach for rating the quality of treatment effect estimates from network meta-analysis. BMJ. 2014;349:g5630. doi:10.1136/bmj.g5630 25252733

[9] Kosuwon W. Treatment of de Quervain tenosynovitis: a prospective randomized controlled study comparing the results of steroid injection with and without immobilization in a splint. J Clin Epidemiol. 1996;49(suppl 1):S5. doi:10.1016/0895-4356(96)89170-0

[10] Heshmati A, Ilka S. Effect of dose of the corticosteroid injected locally on inflammatory diseases. Curr Orthop Pract. 2019;20(2):160-163. doi:10.1097/BCO.0000000000000734

[11] Ansari M. De Quervain’s disease—a randomised prospective study evaluating the efficacy of steroid and conservative management. Int J Pharm Sci Invent. 2014;3(5):4-6.

[12] Avci S, Yilmaz C, Sayli U. Comparison of nonsurgical treatment measures for de Quervain’s disease of pregnancy and lactation. J Hand Surg Am. 2002;27(2):322-324. doi:10.1053/jhsu.2002.32084 11901392

[13] Sharma R, Thukral A, Kumar S, Bhargava SK. Effect of low level lasers in de Quervains tenosynovitis: prospective study with ultrasonographic assessment. Physiotherapy. 2002;88(12):730-734. doi:10.1016/S0031-9406(05)60716-X

[14] Jirarattanaphochai K, Saengnipanthkul S, Vipulakorn K, Jianmongkol S, Chatuparisute P, Jung S. Treatment of de Quervain disease with triamcinolone injection with or without nimesulide: a randomized, double-blind, placebo-controlled trial. J Bone Joint Surg Am. 2004;86(12):2700-2706. doi:10.2106/00004623-200412000-00017 15590856

[15] Goldfarb CA, Gelberman RH, McKeon K, Chia B, Boyer MI. Extra-articular steroid injection: early patient response and the incidence of flare reaction. J Hand Surg Am. 2007;32(10):1513-1520. doi:10.1016/j.jhsa.2007.08.002 18070637

[16] Peters-Veluthamaningal C, Winters JC, Groenier KH, Meyboom-DeJong B. Randomised controlled trial of local corticosteroid injections for de Quervain’s tenosynovitis in general practice. BMC Musculoskelet Disord. 2009;10(1):131. doi:10.1186/1471-2474-10-131 19860883 PMC2774677

[17] Mehdinasab SA, Alemohammad SA. Methylprednisolone acetate injection plus casting versus casting alone for the treatment of de Quervain’s tenosynovitis. Arch Iran Med. 2010;13(4):270-274. 20597558

[18] Pagonis T, Ditsios K, Toli P, Givissis P, Christodoulou A. Improved corticosteroid treatment of recalcitrant de Quervain tenosynovitis with a novel 4-point injection technique. Am J Sports Med. 2011;39(2):398-403. doi:10.1177/0363546510382858 21051423

[19] Abrisham SJ, Karbasi MH, Zare J, Behnamfar Z, Tafti AD, Shishesaz B. De Qeurvian tenosynovitis: clinical outcomes of surgical treatment with longitudinal and transverse incision. Oman Med J. 2011;26(2):91-93. doi:10.5001/omj.2011.23 22043391 PMC3191679

[20] Jongprasitkul H, Suputtitada A, Kitisomprayoonkul W, Pintawiruj K. Elastic bandage vs neoprene thumb stabilizer splint in acute De Quervain’s tenosynovitis. Asian Biomed. 2011;5(2):263-267. doi:10.5372/1905-7415.0502.035

[21] Kume K, Amano K, Yamada S, Amano K, Kuwaba N, Ohta H. In de Quervain’s with a separate EPB compartment, ultrasound-guided steroid injection is more effective than a clinical injection technique: a prospective open-label study. J Hand Surg Eur Vol. 2012;37(6):523-527. doi:10.1177/1753193411427829 22095403

[22] Kang HJ, Koh IH, Jang JW, Choi YR. Endoscopic versus open release in patients with de Quervain’s tenosynovitis: a randomised trial. Bone Joint J. 2013;95-B(7):947-951. doi:10.1302/0301-620X.95B7.31486 23814248

[23] Homayouni K, Zeynali L, Mianehsaz E. Comparison between kinesio taping and physiotherapy in the treatment of de Quervain’s disease. J Musculoskelet Res. 2013;16(4):1350019. doi:10.1142/S021895771350019X

[24] Hadianfard M, Ashraf A, Fakheri M, Nasiri A. Efficacy of acupuncture versus local methylprednisolone acetate injection in De Quervain’s tenosynovitis: a randomized controlled trial. J Acupunct Meridian Stud. 2014;7(3):115-121. doi:10.1016/j.jams.2013.10.003 24929455

[25] Mardani-Kivi M, Karimi Mobarakeh M, Bahrami F, Hashemi-Motlagh K, Saheb-Ekhtiari K, Akhoondzadeh N. Corticosteroid injection with or without thumb spica cast for de Quervain tenosynovitis. J Hand Surg Am. 2014;39(1):37-41. doi:10.1016/j.jhsa.2013.10.013 24315492

[26] Kumar K. Outcome of longitudinal versus transverse incision in de Quervain’s disease and its implications in Indian population. Musculoskelet Surg. 2016;100(1):49-52. doi:10.1007/s12306-015-0388-6 26645452

[27] Orlandi D, Corazza A, Fabbro E, . Ultrasound-guided percutaneous injection to treat de Quervain’s disease using three different techniques: a randomized controlled trial. Eur Radiol. 2015;25(5):1512-1519. doi:10.1007/s00330-014-3515-0 25465711

[28] Tabinda H, Mahmood F. De Quervain’s tenosynovitis and phonophoresis: a randomised controlled trial in pregnant females. J Orthop Trauma Rehabil. 2015;19(1):2-6. doi:10.1016/j.jotr.2014.04.001

[29] Menendez ME, Thornton E, Kent S, Kalajian T, Ring D. A prospective randomized clinical trial of prescription of full-time versus as-desired splint wear for de Quervain tendinopathy. Int Orthop. 2015;39(8):1563-1569. doi:10.1007/s00264-015-2779-6 25916954

[30] Sharma R, Aggarwal AN, Bhatt S, Kumar S, Bhargava SK. Outcome of low level lasers versus ultrasonic therapy in de Quervain’s tenosynovitis. Indian J Orthop. 2015;49(5):542-548. doi:10.4103/0019-5413.164050 26538761 PMC4598546

[31] Awan WA, Babur MN, Masood T. Effectiveness of therapeutic ultrasound with or without thumb spica splint in the management of de Quervain’s disease. J Back Musculoskelet Rehabil. 2017;30(4):691-697. doi:10.3233/BMR-160591 28035912

[32] Lu H, Chen L, Chen Q, Shen H, Jiang S, Yu H. A prospective randomized clinical trial of platelet-rich plasma used in surgery for de Quervain tendinopathy. J Biomater Tissue Eng. 2019;7(12):1344-1348. doi:10.1166/jbt.2017.1703

[33] Abdulkader T, Nadkarni K. Comparison between myofascial release and myofascial taping as an adjunct to conventional occupational therapy in the management of dequervain’s tenosynovitis: a randomized controlled trial. Indian J Occup Therapy. 2019;51(4):145-150. doi:10.4103/ijoth.ijoth_26_19

[34] Kim JH, Yang SW, Ham HJ, Kim JP. Tendon subluxation after surgical release of the first dorsal compartment in de Quervain disease. Ann Plast Surg. 2019;82(6):628-635. doi:10.1097/SAP.0000000000001860 31082847

[35] Ippolito JA, Hauser S, Patel J, Vosbikian M, Ahmed I. Nonsurgical treatment of de Quervain tenosynovitis: a prospective randomized trial. Hand (N Y). 2020;15(2):215-219. doi:10.1177/1558944718791187 30060681 PMC7076607

[36] Akhtar M, Faraz Ul Hassan Shah Gillani S, Nadeem RD, Tasneem M. Methylprednisolone acetate injection with casting versus casting alone for the treatment of de Quervain’s tenosynovitis: a randomized controlled trial. J Pak Med Assoc. 2020;70(8):1314-1318. doi:10.5455/JPMA.293180 32794478

[37] Kumar DR. Management of de Quervain tendinitis using corticosteroid injection (CSI) with or without thumb spica cast (TSC). Eur J Mol Clin Med. 2021;7(8):5635-5640.

[38] Shin YH, Choi SW, Kim JK. Prospective randomized comparison of ultrasonography-guided and blind corticosteroid injection for de Quervain’s disease. Orthop Traumatol Surg Res. 2020;106(2):301-306. doi:10.1016/j.otsr.2019.11.015 31899117

[39] Haghighat S, Vahdatpour B, Ataei E. The effect of extracorporeal shockwave therapy on de Quervain tenosynovitis: a clinical trial. Shiraz E Med J. 2021;22(8):e106559. doi:10.5812/semj.106559

[40] Karlıbel İA, Aksoy MK, Alkan A. Paraffin bath therapy in de Quervain’s tenosynovitis: a single-blind randomized controlled trial. Int J Biometeorol. 2021;65(8):1391-1398. doi:10.1007/s00484-021-02111-2 33675398

[41] Das R, Bimol N, Deb D, Meethal S, Singh Y. Efficacy of thumb abduction orthosis versus local methylprednisolone acetate injection in de Quervain’s tenosynovitis: a randomized controlled trial. J Med Soc. 2021;35(1):35. doi:10.4103/jms.jms_125_20

[42] Başar B, Aybar A, Basar G, Başar H. The effectiveness of corticosteroid injection and splint in diabetic de Quervain’s tenosynovitis patients: a single-blind, randomized clinical consort study. Medicine (Baltimore). 2021;100(35):e27067. doi:10.1097/MD.0000000000027067 34477139 PMC8415982

[43] Bölük Şenlikci H, Odabaşı ÖS, Ural Nazlıkul FG, Nazlıkul H. Effects of local anaesthetics (neural therapy) on pain and hand functions in patients with de Quervain tenosynovitis: a prospective randomised controlled study. Int J Clin Pract. 2021;75(10):e14581. doi:10.1111/ijcp.14581 34185386

[44] Salim B, Ansari MT, Kumar VS, Goyal A, Malhotra R. Is Pulley reconstruction better than pulley release for de Quervain’s tenosynovitis: a double-blind randomized controlled trial. J Wrist Surg. 2021;10(5):377-384. doi:10.1055/s-0041-1725171 34631289 PMC8489986

[45] Jung HS, Baek SH, Lee JS. Is a steroid injection in both compartments more effective than an injection in the extensor pollicis brevis subcompartment alone in patients with de Quervain Disease: a randomized, controlled trial. Clin Orthop Relat Res. 2022;480(4):762-770. doi:10.1097/CORR.0000000000002018 34694249 PMC8923580

[46] Bosman R, Duraku LS, van der Oest MJW, . Surgical treatment outcome of de Quervain’s disease: a systematic review and meta-analysis. Plast Reconstr Surg Glob Open. 2022;10(5):e4305. doi:10.1097/GOX.0000000000004305 35539295 PMC9076451

[47] Beutel BG, Doscher ME, Melone CP Jr. Prevalence of a septated first dorsal compartment among patients with and without de Quervain tenosynovitis: an in vivo anatomical study. Hand (N Y). 2020;15(3):348-352. doi:10.1177/1558944718810864 30428712 PMC7225884

[48] Alemohammad AM, Yazaki N, Morris RP, Buford WL, Viegas SF. Thumb interphalangeal joint extension by the extensor pollicis brevis: association with a subcompartment and de Quervain’s disease. J Hand Surg Am. 2009;34(4):719-723. doi:10.1016/j.jhsa.2008.12.015 19345877

[49] Yuasa K, Kiyoshige Y. Limited surgical treatment of de Quervain’s disease: decompression of only the extensor pollicis brevis subcompartment. J Hand Surg Am. 1998;23(5):840-843. doi:10.1016/S0363-5023(98)80160-3 9763259

[50] Monárrez R, Bains SS, Margalit A, Ingari JV. Partial resection of first dorsal compartment extensor retinaculum in de Quervain’s stenosing tendovaginitis release. Tech Hand Up Extrem Surg. 2023;27(1):14-16. doi:10.1097/BTH.0000000000000402 35686888

[51] Suwannaphisit S, Suwanno P, Fongsri W, Chuaychoosakoon C. Comparison of the effect of ketorolac versus triamcinolone acetonide injections for the treatment of de Quervain’s tenosynovitis: a double-blind randomized controlled trial. BMC Musculoskelet Disord. 2022;23(1):831. doi:10.1186/s12891-022-05784-x 36050704 PMC9434938

[52] Peters-Veluthamaningal C, van der Windt DA, Winters JC, Meyboom-de Jong B. Corticosteroid injection for de Quervain’s tenosynovitis. Cochrane Database Syst Rev. 2009;(3):CD005616. 19588376 10.1002/14651858.CD005616.pub2 PMC12673635

[53] Suwannaphisit S, Chuaychoosakoon C. Effectiveness of surgical interventions for treating de Quervain’s disease: a systematic review and meta-analysis. Ann Med Surg (Lond). 2022;77:103620. doi:10.1016/j.amsu.2022.103620 35638053 PMC9142670

[54] Ashraf MO, Devadoss VG. Systematic review and meta-analysis on steroid injection therapy for de Quervain’s tenosynovitis in adults. Eur J Orthop Surg Traumatol. 2014;24(2):149-157. doi:10.1007/s00590-012-1164-z 23412309

[55] Cavaleri R, Schabrun SM, Te M, Chipchase LS. Hand therapy versus corticosteroid injections in the treatment of de Quervain’s disease: a systematic review and meta-analysis. J Hand Ther. 2016;29(1):3-11. doi:10.1016/j.jht.2015.10.004 26705671

[56] Huisstede BM, Gladdines S, Randsdorp MS, Koes BW. Effectiveness of conservative, surgical, and postsurgical interventions for trigger finger, Dupuytren disease, and de Quervain disease: a systematic review. Arch Phys Med Rehabil. 2018;99(8):1635-1649.e21. doi:10.1016/j.apmr.2017.07.014 28860097

