# Supplementary material for: Management of de Quervain Tenosynovitis: A Systematic Review and Network Meta-Analysis
Source: JAMA Netw Open. 2023 Oct 27;6(10):e2337001. doi: 10.1001/jamanetworkopen.2023.37001 (PMC10611995; doi:10.1001/jamanetworkopen.2023.37001)
Supplement: Supplement 1. — eMethods. eAppendix. Complications eFigure 1. Network Forest Plots of Effectiveness of Interventions for de Quervain Tenosynovitis for Short-Term Pain eFigure 2. Results of Pairwise Meta-Analysis of Short-Term Pain of CSI + Thumb Spica Immobilization vs CSI Alone eFigure 3. Results of Pairwise Meta-Analysis of Mid-Term Pain of CSI + Thumb Spica Immobilization vs CSI Alone eFigure 4. Results of Pairwise Meta-Analysis of Short-Term Pain of US-Guided CSI vs Conventional CSI Alone eFigure 5. Results of Pairwise Meta-Analysis of Mid-Term Total Complications of Transverse vs Longitudinal Skin Incision eFigure 6. Results of Pairwise Meta-Analysis of Mid-Term Superficial Radial Nerve Injury of Transverse vs Longitudinal Skin Incision eFigure 7. Results of Pairwise Meta-Analysis of Mid-Term Scar Hypertrophy of Transverse vs Longitudinal Skin Incision eFigure 8. Network Map of Effectiveness of Interventions for de Quervain Tenosynovitis for Mid-Term Pain eFigure 9. Network Forest Plots of Effectiveness of Interventions for de Quervain Tenosynovitis for Mid-Term Pain eFigure 10. Rank Bars of Effectiveness of Interventions for de Quervain Tenosynovitis for Mid-Term Pain eFigure 11. Network Map of Effectiveness of Interventions for de Quervain Tenosynovitis for Short-Term Function eFigure 12. Network Forest Plots of Effectiveness of Interventions for de Quervain Tenosynovitis for Short-Term Function eFigure 13. Rank Bars of Effectiveness of Interventions for de Quervain Tenosynovitis for Short-Term Function eFigure 14. Network Map of Effectiveness of Interventions for de Quervain Tenosynovitis for Mid-Term Function eFigure 15. Network Forest Plots of Effectiveness of Interventions for de Quervain Tenosynovitis for Mid-Term Function eFigure 16. Rank Bars of Effectiveness of Interventions for de Quervain Tenosynovitis for Mid-Term Function eFigure 17. Comparative Treatment Class Effects for Short-Term Pain eFigure 18. Comparative Treatment Class Effects for Mid-Term Pain eFigure 1 [file jamanetwopen-e2337001-s001.pdf]

# Supplemental Online Content

Challoumas D, Ramasubbu R, Rooney E, Seymour-Jackson E, Putti A, Millar NL. Management of de Quervain tenosynovitis: a systematic review and network meta-analysis. *JAMA Netw Open*. 2023;6(10):e2337001. doi:10.1001/jamanetworkopen.2023.37001

**eMethods.**
**eAppendix.** Complications
**eFigure 1.** Network Forest Plots of Effectiveness of Interventions for de Quervain Tenosynovitis for Short-Term Pain
**eFigure 2.** Results of Pairwise Meta-Analysis of Short-Term Pain of CSI + Thumb Spica Immobilization vs CSI Alone
**eFigure 3.** Results of Pairwise Meta-Analysis of Mid-Term Pain of CSI + Thumb Spica Immobilization vs CSI Alone
**eFigure 4.** Results of Pairwise Meta-Analysis of Short-Term Pain of US-Guided CSI vs Conventional CSI Alone
**eFigure 5.** Results of Pairwise Meta-Analysis of Mid-Term Total Complications of Transverse vs Longitudinal Skin Incision
**eFigure 6.** Results of Pairwise Meta-Analysis of Mid-Term Superficial Radial Nerve Injury of Transverse vs Longitudinal Skin Incision
**eFigure 7.** Results of Pairwise Meta-Analysis of Mid-Term Scar Hypertrophy of Transverse vs Longitudinal Skin Incision
**eFigures 8.** Network Map of Effectiveness of Interventions for de Quervain Tenosynovitis for Mid-Term Pain
**eFigure 9.** Network Forest Plots of Effectiveness of Interventions for de Quervain Tenosynovitis for Mid-Term Pain
**eFigure 10.** Rank Bars of Effectiveness of Interventions for de Quervain Tenosynovitis for Mid-Term Pain
**eFigures 11.** Network Map of Effectiveness of Interventions for de Quervain Tenosynovitis for Short-Term Function
**eFigure 12.** Network Forest Plots of Effectiveness of Interventions for de Quervain Tenosynovitis for Short-Term Function
**eFigure 13.** Rank Bars of Effectiveness of Interventions for de Quervain Tenosynovitis for Short-Term Function
**eFigures 14.** Network Map of Effectiveness of Interventions for de Quervain Tenosynovitis for Mid-Term Function
**eFigure 15.** Network Forest Plots of Effectiveness of Interventions for de Quervain Tenosynovitis for Mid-Term Function
**eFigure 16.** Rank Bars of Effectiveness of Interventions for de Quervain Tenosynovitis for Mid-Term Function
**eFigure 17.** Comparative Treatment Class Effects for Short-Term Pain
**eFigure 18.** Comparative Treatment Class Effects for Mid-Term Pain
**eFigure 19.** Comparative Treatment Class Effects for Short-Term Function
**eFigure 20.** Comparative Treatment Class Effects for Mid-Term Function

© 2023 Challoumas D et al. *JAMA Network Open.*

**eTable 1.** Results of Each Study Showing the Mean Difference Between the Compared Interventions for Pain and Function
**eTable 2.** Results of Risk of Bias Assessment Using the Cochrane ROB 2 Tool
**eTable 3.** Results of Certainty of Evidence Assessment Using the GRADE Tool
**eTable 4.** Summary of Findings for Short-Term Pain
**eTable 5.** Summary of Findings for Mid-Term Pain
**eTable 6.** Summary of Findings for Short-Term Function
**eTable 7.** Summary of Findings for Mid-Term Function

This supplemental material has been provided by the authors to give readers additional information about their work.

**eMethods**

This living systematic review and network meta-analysis (NMA) was prospectively registered on PROSPERO (CRD42022346986), conducted and authored as per PRISMA-NMA" and "PERSiST" guidance5,6. We plan to update the NMA annually for a minimum of 5 years when new eligible data are identified through annual searches and re-perform analyses where necessary. A plain-language summary for patients and clinicians dealing with dQt will also be provided.

**Eligibility Criteria**

**Types of studies**

Randomised Control Trials (RCTs) of any type were eligible for inclusion. Only studies published in English were screened for inclusion.

**Types of participants**
Studies of patients with dQt of any chronicity were eligible for inclusion. A clinical diagnosis of dQt made by a medical professional was required for inclusion with or without radiographic confirmation.No specific diagnostic criteria or tests were used. The present study excluded trials with (1) mixed populations including patients with dQt and other conditions, (2) specific populations with a predisposing condition, e.g. diabetics, pregnant/peripartum women.

**Types of interventions**
Studies assessing the effectiveness of any intervention for dQt were included. No treatment or placebo/sham interventions were also included.

**Types of comparators**
Any intervention, placebo/sham treatment or no treatment.

**Types of outcome measures**
The primary outcome was patient-reported pain, measured by the visual analogue scale (VAS) or equivalent (0-10 or 0-100). Secondary outcomes included patient-reported function and complications. For the purpose of analysis and pooling of results, outcome measures were divided into three distinct intervals, short-term (≤12 weeks), mid-term (>12 weeks– ≤12 months) and long-term (>12 months). Where studies reported results at more than one time points within our pre-specified intervals, those closest to the mid-point of the interval were used.
When trials used different types of patient-reported pain, the following hierarchy was used: a) pain at rest, b) pain with (any) activity, c) tenderness.

**Literature search**
Search strategies were developed in "all fields" with the following Boolean operators: a) '(de Quervain* syndrome OR first dorsal compartment OR first extensor

compartment OR tenosynovitis) AND (treatment OR management OR therapy OR intervention OR injection OR immobilisation OR cast OR splint OR *steroid OR spica OR exercise OR shockwave OR laser OR ultrasound)'.

The following databases were screened for published and unpublished trials from inception to August 2022 by two authors: Medline, Embase, PubMed, Cochrane central, Scopus, OpenGrey.eu and WorldCat.org. For unpublished or ongoing studies, we searched the WHO International Clinical Trials Registry Platform (http://apps.who.int/trialsearch/) Clinical Trials.gov, The European Union Clinical Trials Register and the ISRCTN registry.  The reference lists of all eligible studies were screened for further eligible trials. The PRISMA flowchart is illustrated in Figure 1 (Supplement).  A total of 4467 studies were identified after removal of duplicates. An additional study was identified from reference lists. The titles, abstracts and full texts of the remaining studies were screened for inclusion by two authors independently.

**Data extraction**

Patient characteristics, duration of symptoms, nature of therapeutic intervention(s), outcome measures and follow up time points were extracted from individual trials and recorded in previously constructed data extraction tables in Microsoft Word version 16.43 (Microsoft corp) by two authors separately. For missing data, attempts were made to contact the corresponding authors of studies published in the last 5 years.

**Data Handling – Synthesis of Results**

Comparisons of interventions reported by two or more studies at similar follow up time points were pooled quantitatively by pairwise meta-analyses in the absence of significant clinical heterogeneity (similar populations, follow up time points and interventions). Raw mean differences (MD) and odds ratios (OR) with their accompanying 95% confidence interval (CI) were calculated and used in the tests for continuous and dichotomous outcomes respectively.  Finally, a network meta-analysis was conducted for pain VAS and quick-DASH (q-DASH; functional disability) at each follow up time period where adequate data existed. Whilst a pairwise meta-analysis pools evidence from RCTs that compare the same two interventions and gives direct evidence only, a network meta-analysis extends this to multiple interventions and can rank the included interventions based on their probability of being the most effective using both direct and indirect estimates.

**Protocol deviations**

"Quality of life" and "grip strength" were removed from our secondary outcome measures as they were not included in the majority of RCTs; instead, we included complications as a secondary outcome measure as we thought it would be an important consideration for practice recommendations.

© 2023 Challoumas D et al. *JAMA Network Open.*

## Risk of bias and strength of evidence assessment

The Cochrane Risk of Bias Tool 2 (RoB 2) was used to assess risk of bias for each RCT7. Studies were assessed by two authors separately and controversies were resolved with involvement of the senior author. The overall RoB for each RCT was labelled as "low", "some concerns" or "high" based on the result of the tool's algorithm and the assessor's judgment. RoB was assessed only for studies that participated in quantitative analyses.

The Grading of Recommendations Assessment, Development and Evaluation for network meta-analysis (GRADE-NMA) was used to appraise the certainty (strength) of evidence8. For pairwise meta-analyses, certainty of evidence was assessed based on five domains: overall RoB, imprecision, inconsistency, indirectness and other confounding factors (including publication bias where applicable). The result of each comparison of interventions was assigned one of high, moderate, low or very low certainty of evidence. This process was completed independently by two authors for each outcome measure and disagreements were resolved by involvement of the senior author. The certainty of evidence started from "high" and was downgraded for one step each time when any one of the five domains raised concerns; Where statistical heterogeneity (inconsistency) was found to be high (I2=50-80%), the certainty of evidence was downgraded by one level; where it was found to be substantial (I2>80%), it was downgraded by two levels. In those cases, subgroup analyses were performed to try and explain this heterogeneity only when there were significant differences in populations, interventions, comparators or outcomes of the pooled studies. The certainty of evidence was upgraded when the magnitude of effect was large with both clinical and statistical significance.

For network meta-analyses, the certainty of evidence of the direct estimate was rated first using overall RoB, inconsistency (statistical heterogeneity), indirectness (clinical heterogeneity) and publication bias where applicable. Subsequently, the indirect estimate was rated using the lowest of the ratings of the two direct comparisons forming the most dominant first-order loops and intransitivity (differences in study characteristics of studies used in indirect comparisons). Finally, the network estimate was rated using the highest certainty of evidence between direct and indirect estimates, incoherence (difference between direct and indirect comparisons - assessed using the "node splitting" approach) and imprecision.

Recommendations for clinical practice were strong only when based on results of high or moderate certainty of evidence. The results of pairwise meta-analyses were considered more significant than those of the treatment ranks in network meta-analyses as they are based only on direct comparisons. Clinical significance for each comparison of interventions was defined as a difference equal to or greater than the minimum clinically important difference for each outcome measure. This was defined as 1.4 points for pain VAS and 15 points for q-DASH based on relevant published evidence9,10.

## Statistical analysis

The Review Manager V.5 (RevMan) software was used to calculate pooled MDs with 95% confidence intervals (CI) and generate forest plots for pairwise meta-analyses

© 2023 Challoumas D et al. *JAMA Network Open.*

and their accompanying heterogeneity tests (Chi2 and I2) and P values. STATA 16.1 with Ian White's extension (multivariate random-effects meta-regression) was used for network meta-analyses (frequentist approach)11 ). When exact numerical mean, mean difference or standard deviation (SD) values were not recorded in the individual papers, an estimated value was extrapolated from available graphs. When the results were recorded as mean difference and interquartile range (IQR's) or total range, the SD value was derived as IQR divided by 1.35 or [(max-min)/4] respectively. Where median values were reported, the mean was assumed to be the same. The RevMan software was used to convert CI to SDs. In studies in which only mean values were presented without SDs, the prognostic method described by Ma et al. (2008) was used to generate a SD by calculating the mean of all the other SDs in that comparison12. Statistical significance was set at P<0.05.

The following formula was used for the sample size calculation as part of GRADE's assessment for "imprecision":
N= (2 〖(a+b)〗^2 〖SD〗^2)/x^2
Where:
N=optimal information size
x = MCID; defined as 1.4 points for pain VAS and 15 points for q-DASH (9,10)
SD2=population variance (calculated using pooled SD from included treatment groups, 1.1 points for VAS, 11.8 points for q-DASH)
a = 1.96 (for 5% type I error)
b = 0.842 (for 80% power)

The optimal information size ("N"; minimum number of overall patients combined in each meta-analysis for sufficient "precision" in the GRADE assessment) with the use of the above formula was calculated as 20 patients both for pain VAS and quick DASH. Potential publication bias was not assessed as no pairwise meta-analyses included more than 10 studies. Expecting wide-range variability in studies' settings, a random-effects model was employed in all meta-analyses.

**eAppendix.** Complications
Other than complications related to transverse and longitudinal incision, quantitative analyses were not possible for all other data, therefore the results of the relevant studies are only presented qualitatively. A total of 9 studies[14,18-21,25,39,42,49] that involved CSI as a treatment for dQt assessed for complications. Four (4) reported no complications. In the remaining 5 studies, skin hypopigmentation ranged from 1-79% and transient post-injection increase in pain from 14-41%. A study[18] also demonstrated a 3% incidence of subcutaneous nodules and ecchymosis at the injection site.

**eFigure 1.** Network forest plots of effectiveness of interventions for de Quervain's tenosynovitis for short-term (0-12 weeks) pain. In fig. 8, the diameter of the circle represents the number of studies assessing that intervention and the thickness of the line represents the number of studies assessing that comparison of interventions. *CSI, conventional corticosteroid injection; EPB, extensor pollicis brevis; ESWT, extracorporeal shockwave therapy; US, ultrasound.*

© 2023 Challoumas D et al. *JAMA Network Open.*

**eFigure 2**. Results of pairwise meta-analysis of short-term (0-12 weeks) pain of CSI + thumb spica immobilisation vs CSI alone showing forest plot, mean difference with 95% confidence interval, statistical heterogeneity tests and p value. *CSI, corticosteroid injection.*

**eFigure 3.** Results of pairwise meta-analysis of mid-term (13 weeks-12 months) pain of CSI + thumb spica immobilisation vs CSI alone showing forest plot, mean difference with 95% confidence interval, statistical heterogeneity tests and p value. *CSI, corticosteroid injection.*

**eFigure 4**. Results of pairwise meta-analysis of short-term (0-12 weeks) pain of US-guided CSI vs conventional CSI alone showing forest plot, mean difference with 95% confidence interval, statistical heterogeneity tests and p value. *CSI, corticosteroid injection; US, ultrasound.*

**eFigure 5.** Results of pairwise meta-analysis of mid-term (13 weeks-12 months) total complications of transverse vs longitudinal skin incision (open surgery) showing forest plot, odds ratio with 95% confidence interval, statistical heterogeneity tests and p value. *CSI, corticosteroid injection.*

**eFigure 6.** Results of pairwise meta-analysis of mid-term (13 weeks-12 months) superficial radial nerve injury of transverse vs longitudinal skin incision (open surgery) showing forest plot, odds ratio with 95% confidence interval, statistical heterogeneity tests and p value. *CSI, corticosteroid injection.*

**eFigure 7.** Results of pairwise meta-analysis of mid-term (13 weeks-12 months) scar hypertrophy of transverse vs longitudinal skin incision (open surgery) showing forest plot, odds ratio with 95% confidence interval, statistical heterogeneity tests and p value. *CSI, corticosteroid injection.*

**eFigures 8-10**. Network map (fig. 8), network forest plots (fig. 9) and rank bars (fig. 10) of effectiveness of interventions for de Quervain's tensosynovitis for mid-term (13 weeks - 12 months) pain. In fig. 11, the diameter of the circle represents the number of studies assessing that intervention and the thickness of the line represents the number of studies assessing that comparison of intereventions.
A, Conventional CSI; B, Conventional CSI + thumb spica immobilisation; C, Thumb spica splint (full-time wear); D, Thumb spica cast; E, Neural therapy + thumb spica cast; F, US-guided CSI (both compartments); G, US-guided CSI (EPB compartment only).
*CSI, conventional corticosteroid injection; EPB, extensor pollicis brevis; US, ultrasound*

**eFigures 11-13**. Network map (fig. 11), network forest plots (fig. 12) and rank bars (fig. 13) of effectiveness of interventions for de Quervain's tensosynovitis for short-term (0-12 weeks) function (q-DASH score). In fig. 11, the diameter of the circle represents the number of studies assessing that intervention and the thickness of the line represents the number of studies assessing that comparison of interventions.

© 2023 Challoumas D et al. *JAMA Network Open.*

A, Conventional CSI; B, Conventional CSI + thumb spica immobilisation; C, Thumb spica splint (full-time wear); D, ESWT + thumb spica splint; E, Thumb spica cast; F, As-decided thumb spica splint wear; G, Acupuncture.
*CSI, conventional corticosteroid injection; US, ultrasound; ESWT, extracorporeal shockwave therapy*

**eFigures 14-16**. Network map (fig. 14), network forest plots (fig. 15) and rank bars (fig. 16) of effectiveness of interventions for de Quervain's tensosynovitis for mid-term (13 weeks - 12 months) function (q-DASH score). In fig. 14, the diameter of the circle represents the number of studies assessing that intervention and the thickness of the line represents the number of studies assessing that comparison of interventions.
A, Conventional CSI; B, Conventional CSI + thumb spica immobilisation; C, Thumb spica splint (full-time wear). *CSI, corticosteroid injection*

**eFigure 17.** Comparative treatment class effects expressed as coefficient with 95% CI for short-term (0-12 weeks) pain VAS. Each value represents the result of the comparison of the intervention of that column vs the intervention of that row. A negative value favours the column intervention and a positive value the row intervention. 95% CIs that do not include 0 denote statistical significance. The last row shows the median rank with 95% confidence intervals for the intervention of that column.
*CSI, conventional corticosteroid injection; EPB, extensor pollicis brevis; ESWT, extracorporeal shockwave therapy; HA, hyaluronic acid; US, ultrasound*

**eFigure 18.** Comparative treatment class effects expressed as coefficient with 95% CI for mid-term (13 weeks – 12 months) pain VAS. Each value represents the result of the comparison of the intervention of that column vs the intervention of that row. A negative value favours the column intervention and a positive value the row intervention. 95% CIs that do not include 0 denote statistical significance. The last row shows the median rank with 95% confidence intervals for the intervention of that column. *CSI, conventional corticosteroid injection; EPB, extensor pollicis brevis; US, ultrasound*

**eFigure 19.** Comparative treatment class effects expressed as coefficient with 95% CI for short-term (0-12 weeks) function (qDASH). Each value represents the result of the comparison of the intervention of that column vs the intervention of that row. A negative value favours the column intervention and a positive value the row intervention. 95% CIs that do not include 0 denote statistical significance. The last row shows the median rank with 95% confidence intervals for the intervention of that column.
*CSI, conventional corticosteroid injection; ESWT, extracorporeal shockwave therapy*

**eFigure 20.** Comparative treatment class effects expressed as coefficient with 95% CI for mid-term (13 weeks – 12 months) function (qDASH). Each value represents the result of the comparison of the intervention of that column vs the intervention of that row. A negative value favours the column intervention and a positive value the row intervention. 95% CIs that do not include 0 denote statistical significance. The last row

© 2023 Challoumas D et al. *JAMA Network Open.*

shows the median rank with 95% confidence intervals for the intervention of that column

**eTable 1.** Results of each study showing the mean difference between the compared interventions for pain and function. The up and down arrows represent statistical significance. Up arrow and positive value for both pain and function favours intervention 1, down arrow and negative value favours intervention 2.
*CSI, corticosteroid injection; ESWT, extracorporeal shockwave therapy; HA, hyaluronic acid; MT, mid-term; PRP, platelet-rich plasma; ST, short-term; US, ultrasound*

**eTable 2.** Results of risk of bias assessment using the Cochrane RoB 2 tool. *RoB, risk of bias*

**eTable 3.** Results of certainty of evidence assessment using the GRADE tool. For inconsistency, the certainty of evidence was downgraded by one step where statistical heterogeneity was significant ($I^2$=50-80%) and by two steps where it was substantial ($I^2$>80%). For imprecision, the certainty of evidence was downgraded by one step if the confidence interval was wide or the optimal information size was not met and by two steps where the confidence interval was very wide. The results of all comparisons were downgraded by one step for overall risk of bias and there was no downgrading for indirectness (clinical heterogeneity) or “other” as publication bias was not assessed formally (no comparisons included more than ten studies).

**eTable 4.** Summary of findings table for short-term (0-12 weeks) pain VAS. In the interpretation of findings column, an intervention was “definitely” (instead of probably) superior or inferior to the reference intervention when both statistical and clinical significance were reached and the certainty of evidence was at least moderate.
[B]: Downgraded due to overall risk of bias
[I]: downgraded due to inconsistency
*: upgraded due to large magnitude of effect
*CSI, conventional corticosteroid injection; EPB, extensor pollicis brevis; ESWT, extracorporeal shockwave therapy; HA, hyaluronic acid; RCT, randomised clinical trial; US, ultrasound*

**eTable 5.** Summary of findings table for mid-term (13 weeks – 12 months) pain VAS. In the interpretation of findings column, an intervention was “definitely” (instead of probably) superior or inferior to the reference intervention when both statistical and clinical significance were reached and the certainty of evidence was at least moderate
[B]: Downgraded due to overall risk of bias
[I]: downgraded due to inconsistency
*CSI, conventional corticosteroid injection; EPB, extensor pollicis brevis; RCT, randomised clinical trial; US, ultrasound*

© 2023 Challoumas D et al. *JAMA Network Open.*

**eTable 6.** Summary of findings table for short-term (0-12 weeks) function qDASH. In the interpretation of findings column, an intervention was "definitely" (instead of probably) superior or inferior to the reference intervention when both statistical and clinical significance were reached and the certainty of evidence was at least moderate.
[B]: Downgraded due to overall risk of bias
*: upgraded due to large magnitude of effect
*CSI, conventional corticosteroid injection; ESWT, extracorporeal shockwave therapy; RCT, randomised clinical trial.*

**eTable 7.** Summary of findings table for mid-term (13 weeks – 12 months) function qDASH. In the interpretation of findings column, an intervention was "definitely" (instead of probably) superior or inferior to the reference intervention when both statistical and clinical significance were reached and the certainty of evidence was at least moderate.
[B]: Downgraded due to overall risk of bias
*CSI, conventional corticosteroid injection; RCT, randomised clinical trial*

© 2023 Challoumas D et al. *JAMA Network Open.*

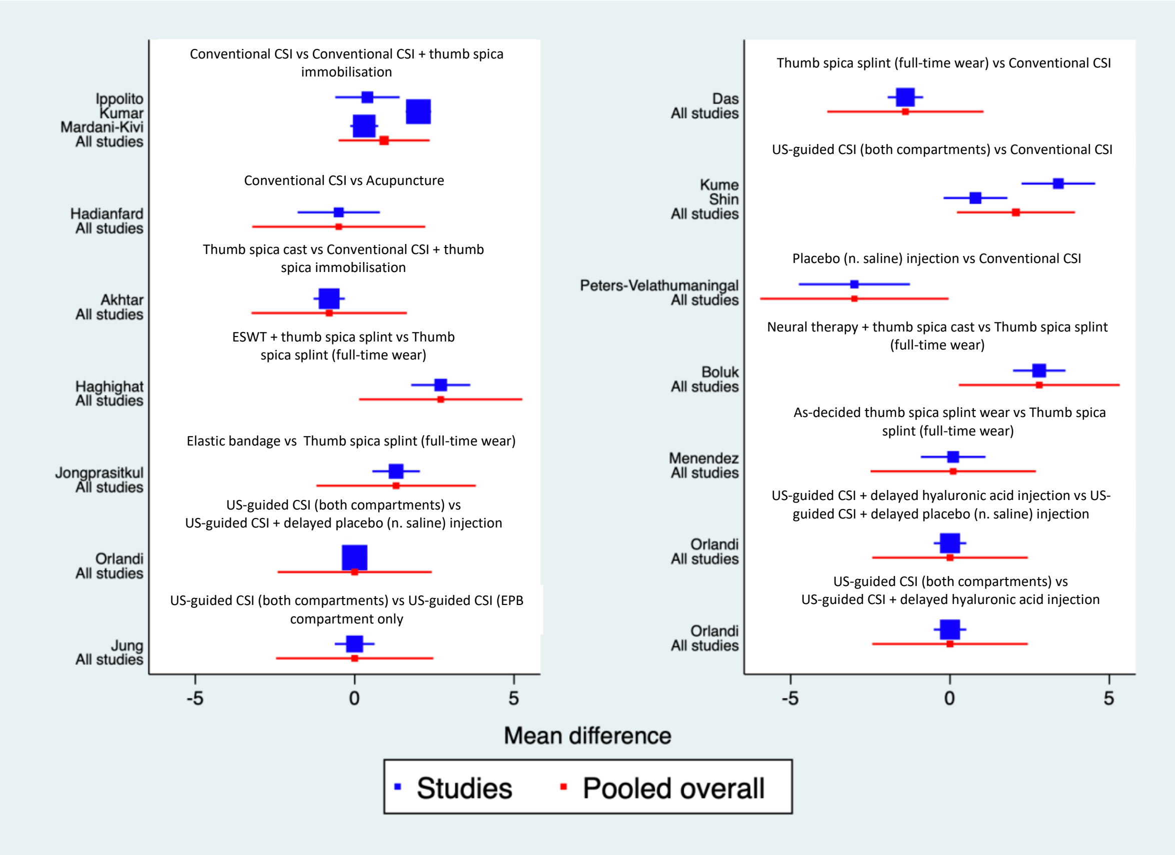


**eFigure 1.** Network forest plots of effectiveness of interventions for de Quervain's tenosynovitis for short-term (0-12 weeks) pain. In fig. 8, the diameter of the circle represents the number of studies assessing that intervention and the thickness of the line represents the number of studies assessing that comparison of interventions. *CSI, conventional corticosteroid injection; EPB, extensor pollicis brevis; ESWT, extracorporeal shockwave therapy; US, ultrasound.*

© 2023 Challoumas D et al. *JAMA Network Open.*

| Study or Subgroup | CSI + thumb spica Mean | SD | Total | CSI Mean | SD | Total | Weight | Mean Difference IV, Random, 95% CI |
|---|---|---|---|---|---|---|---|---|
| Basar 2021 | 7.4 | 0.7 | 41 | 6.6 | 1 | 41 | 26.2% | 0.80 [0.43, 1.17] |
| Ippolito 2020 | 5.4 | 1.2 | 11 | 5.4 | 0.6 | 9 | 22.8% | 0.00 [−0.81, 0.81] |
| Kumar 2020 | 9.3 | 0.8 | 60 | 6.8 | 1.6 | 60 | 25.7% | 2.50 [2.05, 2.95] |
| Mardani-Kivi 2014 | 8.4 | 0.7 | 33 | 6.9 | 1.3 | 34 | 25.4% | 1.50 [1.00, 2.00] |
| Total (95% CI) | | | 145 | | | 144 | 100.0% | 1.23 [0.28, 2.19] |

Heterogeneity: $Tau^2 = 0.87$; $Chi^2 = 44.12$, df = 3 ($P < 0.00001$); $I^2 = 93\%$
Test for overall effect: Z = 2.53 (P = 0.01)

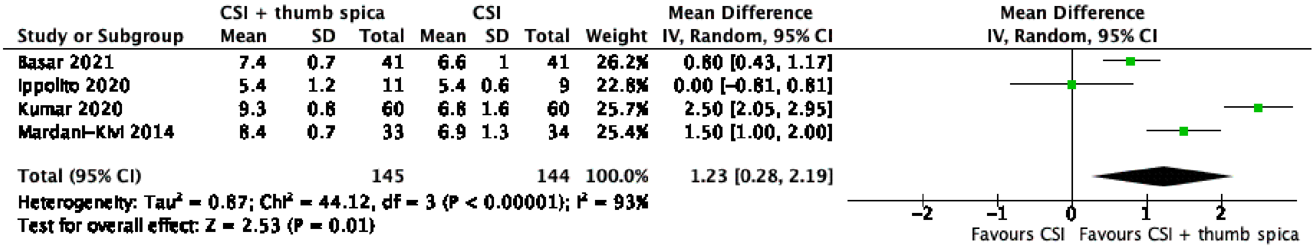


**eFigure 2**. Results of pairwise meta-analysis of short-term (0-12 weeks) pain of CSI + thumb spica immobilisation vs CSI alone showing forest plot, mean difference with 95% confidence interval, statistical heterogeneity tests and p value. *CSI, corticosteroid injection.*

| Study or Subgroup | Transverse incision Events | Total | Longitudinal incision Events | Total | Weight | Odds Ratio M-H, Random, 95% CI |
|---|---|---|---|---|---|---|
| Abrisham 2011 | 3 | 60 | 0 | 60 | 50.6% | 7.37 [0.37, 145.75] |
| Kumar 2015 | 3 | 24 | 0 | 24 | 49.4% | 7.98 [0.39, 163.33] |
| Total (95% CI) | | 84 | | 84 | 100.0% | 7.66 [0.92, 64.00] |
| Total events | 6 | | 0 | | | |

Heterogeneity: $Tau^2 = 0.00$; $Chi^2 = 0.00$, df = 1 ($P = 0.97$); $I^2 = 0\%$
Test for overall effect: Z = 1.88 (P = 0.06)

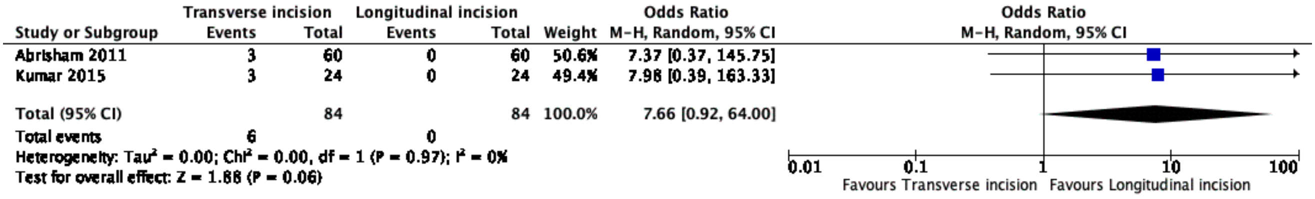


**eFigure 3.** Results of pairwise meta-analysis of mid-term (13 weeks-12 months) pain of CSI + thumb spica immobilisation vs CSI alone showing forest plot, mean difference with 95% confidence interval, statistical heterogeneity tests and p value. *CSI, corticosteroid injection.*

| Study or Subgroup | Conventional CSI Mean | SD | Total | US-guided CSI Mean | SD | Total | Weight | Mean Difference IV, Random, 95% CI |
|---|---|---|---|---|---|---|---|---|
| Kume 2012 | 2 | 2.1 | 22 | 5.4 | 1.8 | 22 | 49.4% | −3.40 [−4.56, −2.24] |
| Shin 2020 | 4.4 | 2 | 24 | 5.2 | 1.5 | 24 | 50.6% | −0.80 [−1.80, 0.20] |
| Total (95% CI) | | | 46 | | | 46 | 100.0% | −2.08 [−4.63, 0.46] |

Heterogeneity: $Tau^2 = 3.08$; $Chi^2 = 11.12$, df = 1 ($P = 0.0009$); $I^2 = 91\%$
Test for overall effect: Z = 1.60 (P = 0.11)

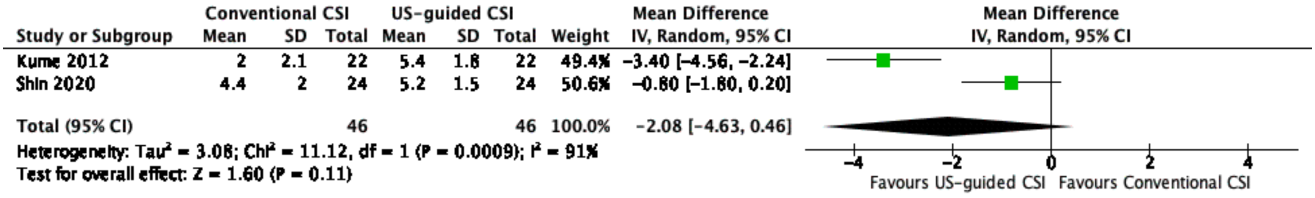


**eFigure 4**. Results of pairwise meta-analysis of short-term (0-12 weeks) pain of US-guided CSI vs conventional CSI alone showing forest plot, mean difference with 95% confidence interval, statistical heterogeneity tests and p value. *CSI, corticosteroid injection; US, ultrasound.*

| Study or Subgroup | Transverse incision Events | Total | Longitudinal incision Events | Total | Weight | Odds Ratio M-H, Random, 95% CI |
|---|---|---|---|---|---|---|
| Abrisham 2011 | 5 | 60 | 5 | 60 | 63.0% | 1.00 [0.27, 3.65] |
| Kumar 2015 | 5 | 24 | 1 | 24 | 37.0% | 6.05 [0.65, 56.37] |
| Total (95% CI) | | 84 | | 84 | 100.0% | 1.95 [0.35, 10.84] |
| Total events | 10 | | 6 | | | |

Heterogeneity: $Tau^2 = 0.78$; $Chi^2 = 1.90$, df = 1 ($P = 0.17$); $I^2 = 47\%$
Test for overall effect: Z = 0.76 (P = 0.45)

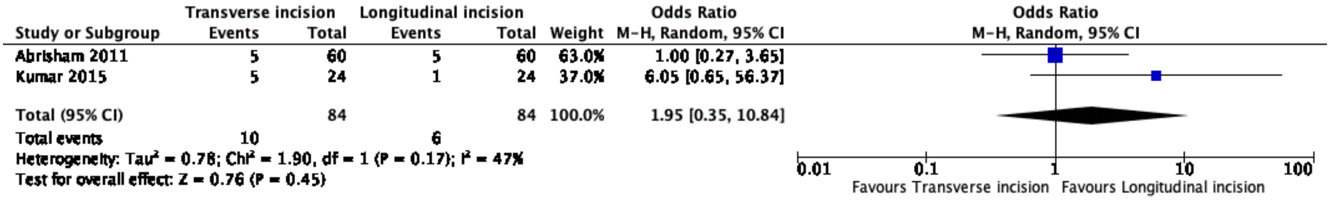


**eFigure 5.** Results of pairwise meta-analysis of mid-term (13 weeks-12 months) total complications of transverse vs longitudinal skin incision (open surgery) showing forest plot, odds ratio with 95% confidence interval, statistical heterogeneity tests and p value. *CSI, corticosteroid injection.*

| Study or Subgroup | Transverse incision Events | Total | Longitudinal incision Events | Total | Weight | Odds Ratio M-H, Random, 95% CI |
|---|---|---|---|---|---|---|
| Abrisham 2011 | 3 | 60 | 0 | 60 | 50.6% | 7.37 [0.37, 145.75] |
| Kumar 2015 | 3 | 24 | 0 | 24 | 49.4% | 7.98 [0.39, 163.33] |
| Total (95% CI) | | 84 | | 84 | 100.0% | 7.66 [0.92, 64.00] |
| Total events | 6 | | 0 | | | |

Heterogeneity: $Tau^2 = 0.00$; $Chi^2 = 0.00$, df = 1 ($P = 0.97$); $I^2 = 0\%$
Test for overall effect: Z = 1.88 (P = 0.06)

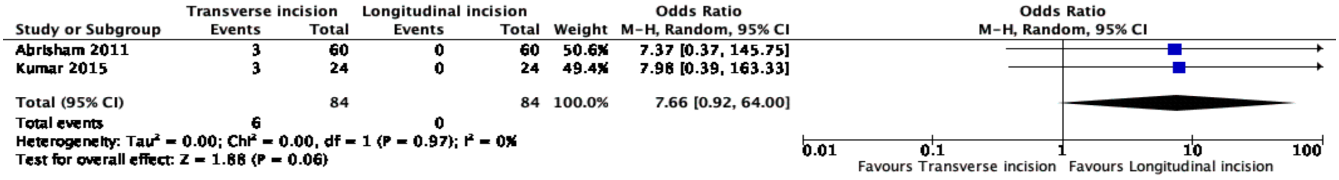


© 2023 Challoumas D et al. *JAMA Network Open.*

**eFigure 6.** Results of pairwise meta-analysis of mid-term (13 weeks-12 months) superficial radial nerve injury of transverse vs longitudinal skin incision (open surgery) showing forest plot, odds ratio with 95% confidence interval, statistical heterogeneity tests and p value. *CSI, corticosteroid injection.*

| | Transverse incision | | Longitudinal incision | | | Odds Ratio |
|---|---|---|---|---|---|---|
| Study or Subgroup | Events | Total | Events | Total | Weight | M–H, Random, 95% CI |
| Abrisham 2011 | 13 | 60 | 5 | 60 | 60.6% | 3.04 [1.01, 9.16] |
| Kumar 2015 | 12 | 24 | 1 | 24 | 39.4% | 23.00 [2.66, 198.66] |
| Total (95% CI) | | 84 | | 84 | 100.0% | 6.75 [0.95, 48.07] |
| Total events | 25 | | 6 | | | |

Heterogeneity: $Tau^2 = 1.34$; $Chi^2 = 2.75$, df = 1 (P = 0.10); $I^2 = 64\%$
Test for overall effect: Z = 1.91 (P = 0.06)

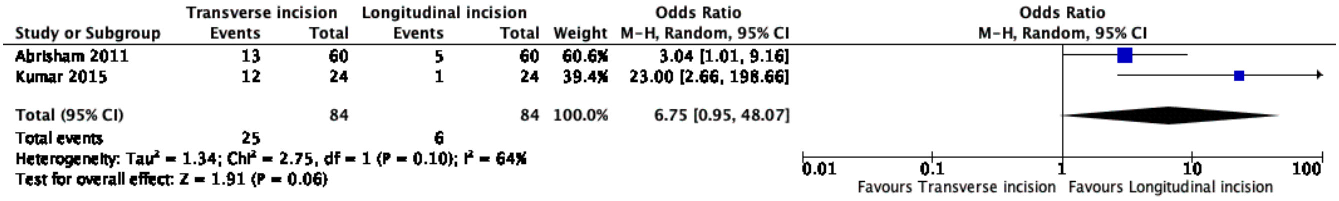


**eFigure 7.** Results of pairwise meta-analysis of mid-term (13 weeks-12 months) scar hypertrophy of transverse vs longitudinal skin incision (open surgery) showing forest plot, odds ratio with 95% confidence interval, statistical heterogeneity tests and p value. *CSI, corticosteroid injection.*

© 2023 Challoumas D et al. *JAMA Network Open.*

8

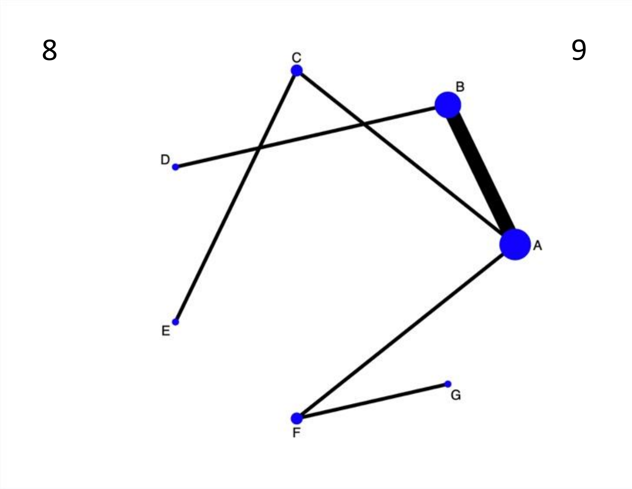


9

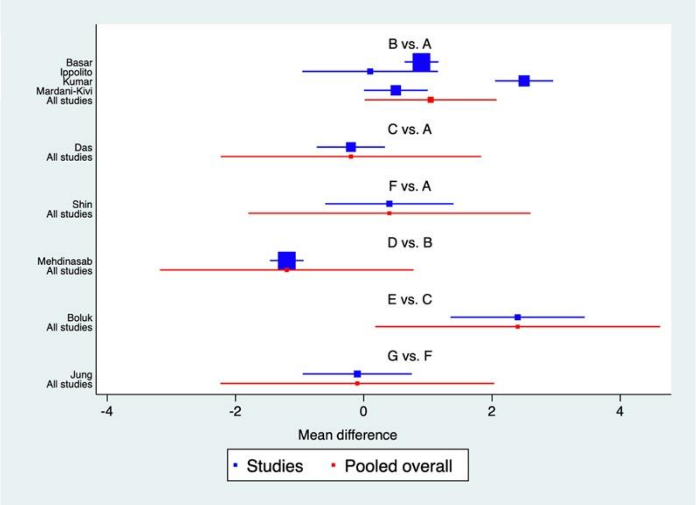


10

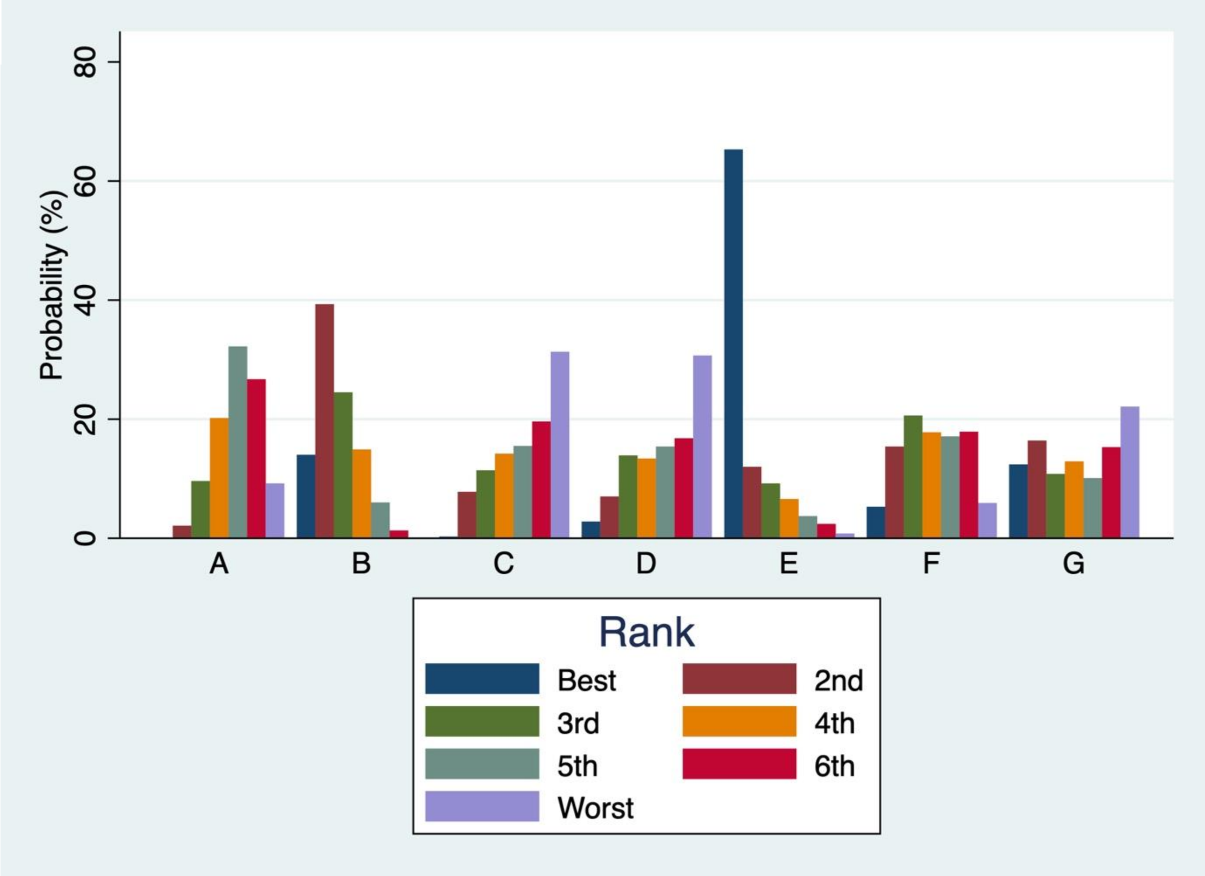


**eFigures 8-10**. Network map (fig. 8), network forest plots (fig. 9) and rank bars (fig. 10) of effectiveness of interventions for de Quervain's tensosynovitis for mid-term (13 weeks - 12 months) pain. In fig. 11, the diameter of the circle represents the number of studies assessing that intervention and the thickness of the line represents the number of studies assessing that comparison of intereventions.

A, Conventional CSI; B, Conventional CSI + thumb spica immobilisation; C, Thumb spica splint (full-time wear); D, Thumb spica cast; E, Neural therapy + thumb spica cast; F, US-guided CSI (both compartments); G, US-guided CSI (EPB compartment only).

*CSI, conventional corticosteroid injection; EPB, extensor pollicis brevis; US, ultrasound*

© 2023 Challoumas D et al. *JAMA Network Open.*

11

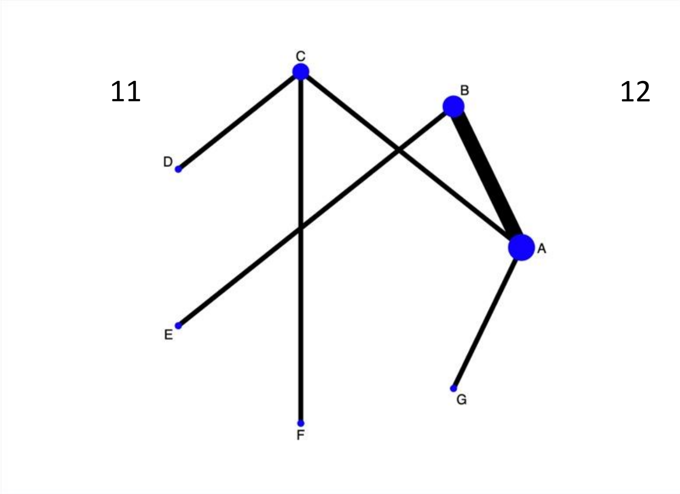


12

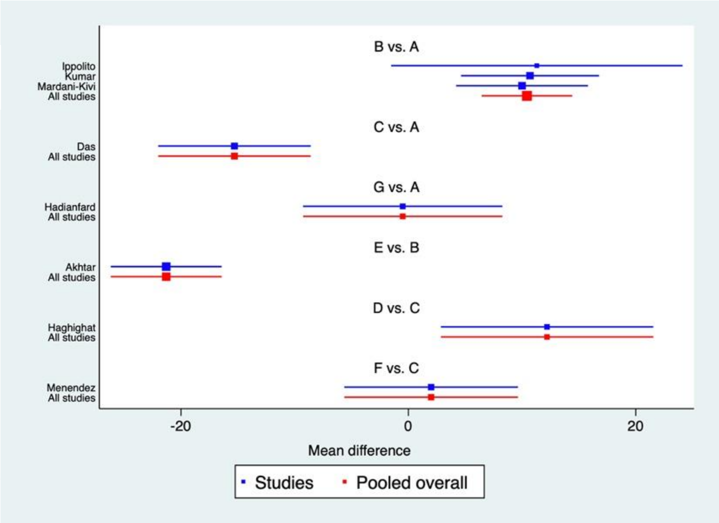


13

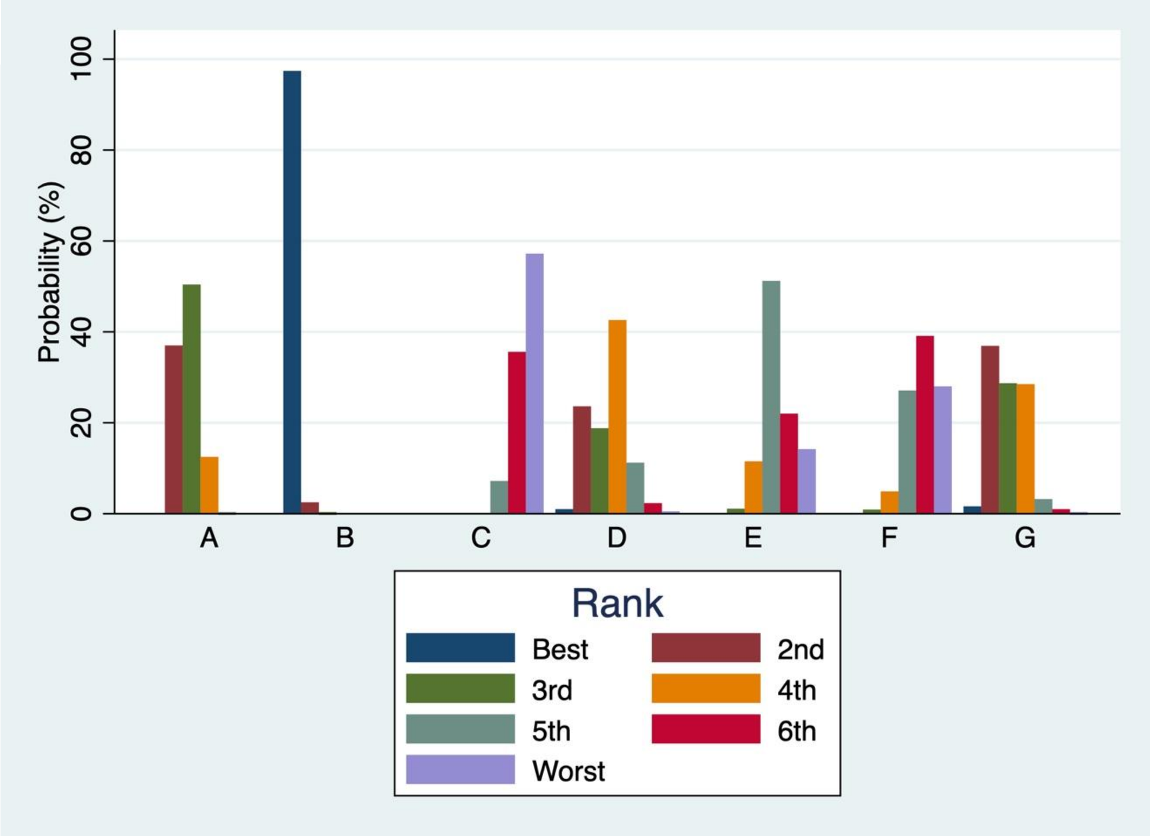


**eFigures 11-13**. Network map (fig. 11), network forest plots (fig. 12) and rank bars (fig. 13) of effectiveness of interventions for de Quervain's tensosynovitis for short-term (0-12 weeks) function (q-DASH score). In fig. 11, the diameter of the circle represents the number of studies assessing that intervention and the thickness of the line represents the number of studies assessing that comparison of interventions.

A, Conventional CSI; B, Conventional CSI + thumb spica immobilisation; C, Thumb spica splint (full-time wear); D, ESWT + thumb spica splint; E, Thumb spica cast; F, As-decided thumb spica splint wear; G, Acupuncture.

*CSI, conventional corticosteroid injection; US, ultrasound; ESWT, extracorporeal shockwave therapy*

© 2023 Challoumas D et al. *JAMA Network Open.*

14

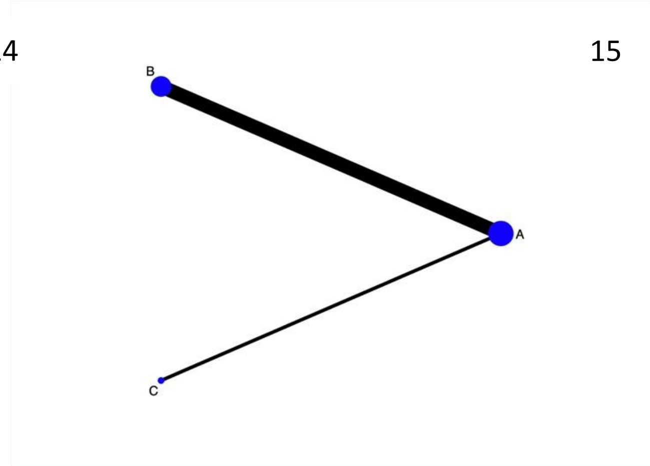


15

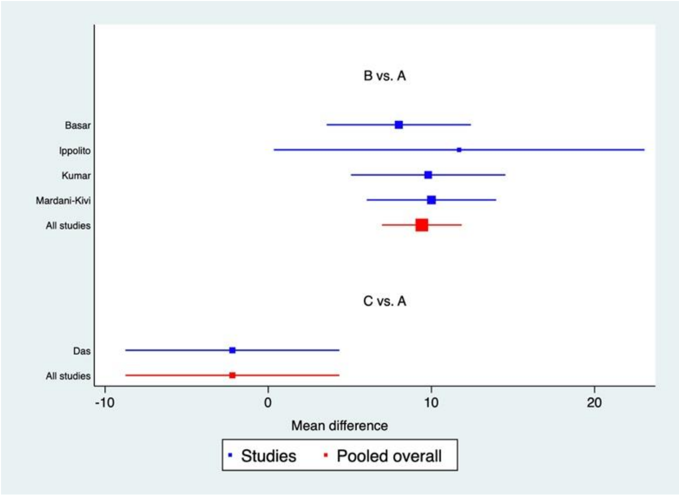


16

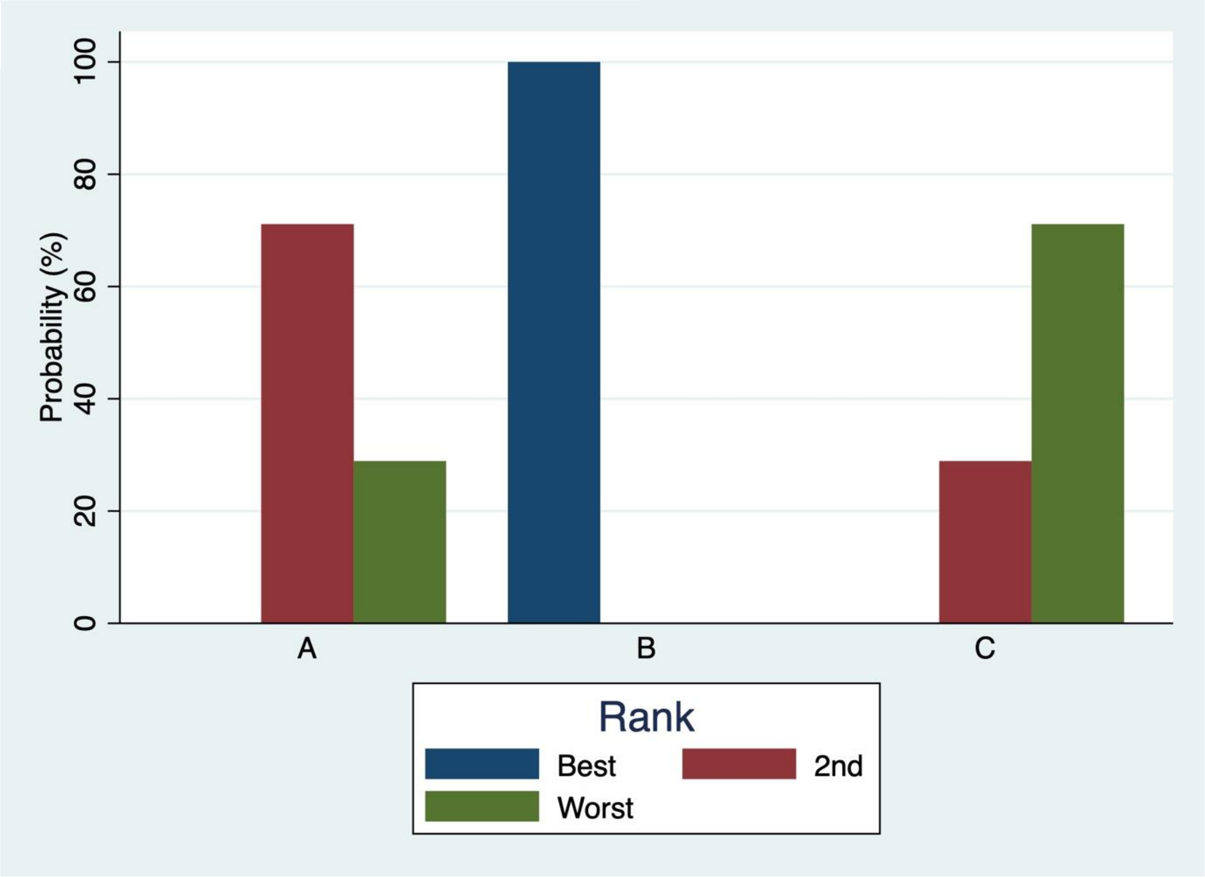


**eFigures 14-16**. Network map (fig. 14), network forest plots (fig. 15) and rank bars (fig. 16) of effectiveness of interventions for de Quervain's tensosynovitis for mid-term (13 weeks - 12 months) function (q-DASH score). In fig. 14, the diameter of the circle represents the number of studies assessing that intervention and the thickness of the line represents the number of studies assessing that comparison of interventions.
A, Conventional CSI; B, Conventional CSI + thumb spica immobilisation; C, Thumb spica splint (full-time wear). *CSI, corticosteroid injection*

© 2023 Challoumas D et al. *JAMA Network Open.*

| | | | | | | | | | | | | | |
|---|---|---|---|---|---|---|---|---|---|---|---|---|---|
| **Conventional CSI** | | | | | | | | | | | | | |
| 0.9 (-0.5 to 2.4) | **Conventional CSI+thumb spica** | | | | | | | | | | | | |
| -1.4 (-3.3 to 0.5) | -2.3 (-5.2 to 0.5) | **Thumb spica splint** | | | | | | | | | | | |
| 2.1 (-1.0 to 5.1) | 1.1 (-2.2 to 4.5) | 3.5 (-0.4 to 7.4) | **US-guided CSI+ n. saline inj** | | | | | | | | | | |
| 2.1 (-1.0 to 5.1) | 1.1 (-2.2 to 4.5) | 3.5 (-0.4 to 7.4) | 0 (-1.9 to 1.9) | **US-guided CSI+HA inj** | | | | | | | | | |
| 1.4 (-1.4 to 4.2) | 0.5 (-2.3 to 3.5) | 2.8 (0.8 to 4.8) | -0.7 (-5.3 to 4.0) | -0.7 (-5.3 to 4.0) | **Neural therapy+thumb spica splint** | | | | | | | | |
| 1.3 (-1.5 to 4.1) | 0.4 (-2.7 to 3.4) | 2.7 (0.6 to 4.8) | -0.8 (-5.4 to 3.9) | -0.8 (-5.4 to 3.9) | -0.1 (-3.0 to 2.8) | **ESWT+thumb spica splint** | | | | | | | |
| 0.1 (-2.1 to 2.4) | -0.8 (-2.7 to 1.1) | 1.5 (-2.2 to 5.3) | -1.9 (-6.1 to 2.2) | -1.9 (-6.1 to 2.2) | -1.2 (-4.9 to 2.3) | -1.2 (-4.7 to 2.4) | **Thumb spica cast** | | | | | | |
| -1.3 (-4.1 to 1.5) | -2.2 (-5.3 to 0.8) | 0.1 (-2.0 to 2.2) | -3.4 (-8.1 to 1.3) | -3.4 (-8.1 to 1.3) | -2.7 (-5.6 to 0.2) | -2.6 (-5.5 to 0.3) | -1.4 (-5.0 to 2.2) | **As-decided thumb spica splint wear** | | | | | |
| 2.1 (-1.0 to 5.2) | 1.1 (-2.3 to 4.5) | 3.5 (-0.4 to 7.4) | 0 (-2.7 to 2.7) | 0 (-2.7 to 2.7) | 0.7 (-3.3 to 4.6) | 0.8 (-3.9 to 5.5) | 1.9 (-2.2 to 6.1) | 3.4 (-1.4 to 8.1) | **US-guided CSI in EPB comp** | | | | |
| -0.5 (-2.7 to 1.7) | -1.4 (-4.0 to 1.1) | 0.9 (-2.1 to 3.9) | -2.6 (-6.6 to 1.5) | -2.6 (-6.6 to 1.5) | -1.9 (-5.5 to 1.7) | -1.8 (-5.4 to 1.8) | -0.6 (-3.8 to 2.5) | 0.8 (-2.8 to 4.4) | -2.6 (-6.7 to 1.5) | **Acupuncture** | | | |
| -0.1 (-2.9 to 2.7) | -1.0 (-4.0 to 2.0) | 1.3 (-0.7 to 1.3) | -2.2 (-6.8 to 2.2) | -2.2 (-6.8 to 2.2) | -1.5 (-4.3 to 1.3) | -1.4 (-4.3 to 1.5) | -0.2 (-3.8 to 3.3) | 1.2 (-1.7 to 4.1) | -2.2 (-6.8 to 2.5) | 0.4 (-3.2 to 4.0) | **Elastic bandage** | | |
| 2.1 (0.2 to 3.9) | 1.1 (-1.2 to 3.5) | 3.5 (0.4 to 6.5) | 0 (-1.9 to 1.9) | 0 (-1.9 to 1.9) | 0.7 (-3.3 to 4.6) | 0.8 (-3.2 to 4.8) | 1.9 (-1.4 to 5.3) | 3.4 (-0.7 to 7.4) | 0 (-1.9 to 1.9) | 2.6 (-0.7 to 5.9) | 2.2 (-1.8 to 6.1) | **US-guided CSI** | |
| -3.0 (-6.0 to -0.1) | -3.9 (-6.7 to -1.2) | -1.6 (-4.8 to -1.6) | -5.1 (-9.3 to -0.8) | -5.1 (-9.3 to -0.8) | -4.4 (-8.2 to -0.6) | -4.3 (-8.9 to -0.3) | -3.1 (-7.2 to 0.2) | -1.7 (-5.5 to 2.1) | -5.1 (-9.3 to -0.8) | -2.5 (-5.9 to 0.9) | -2.9 (-6.7 to 0.9) | -5.1 (-8.6 to -1.6) | **Placebo injection** |

© 2023 Challoumas D et al. *JAMA Network Open.*

| | | | | | | | | | | | | | |
|---|---|---|---|---|---|---|---|---|---|---|---|---|---|
| ***9 (6-12)*** | ***7 (2-11)*** | ***12 (9-14)*** | ***1 (1-7)*** | ***1 (1-7)*** | ***1 (1-10)*** | ***1 (1-10)*** | ***10 (5-13)*** | ***13 (8-14)*** | ***1 (1-6)*** | ***10 (5-14)*** | ***11 (5-13)*** | ***3 (1-6)*** | ***14 (11-14)*** |

**eFigure 17.** Comparative treatment class effects expressed as coefficient with 95% CI for short-term (0-12 weeks) pain VAS. Each value represents the result of the comparison of the intervention of that column vs the intervention of that row. A negative value favours the column intervention and a positive value the row intervention. 95% CIs that do not include 0 denote statistical significance. The last row shows the median rank with 95% confidence intervals for the intervention of that column.
*CSI, conventional corticosteroid injection; EPB, extensor pollicis brevis; ESWT, extracorporeal shockwave therapy; HA, hyaluronic acid; US, ultrasound*

| | | | | | | |
|---|---|---|---|---|---|---|
| **Conventional CSI** | | | | | | |
| **1.0 (0.1,2.1)** | **Conventional CSI+thumb spica** | | | | | |
| -0.2 (-2.2 to 1.8) | -1.2 (-3.5 to 1.0) | **Thumb spica splint** | | | | |
| -0.2 (-2.4 to 2.1) | -1.2 (-3.2 to 0.8) | 0 (-3.0 to 3.1) | **Thumb spica cast** | | | |
| 2.2 (-0.8 to 5.2) | 1.2 (-2.0 to 4.3) | 2.4 (0.2 to 4.6) | 2.4 (-1.4 to 6.1) | **Neural therapy+thumb spica splint** | | |
| 0.4 (-1.8 to 2.6) | -0.6 (-3.1 to 1.8) | 0.6 (-2.4 to 3.6) | 0.6 (-2.6 to 3.7) | -1.8 (-5.5 to 1.9) | **US-guided CSI** | |
| 0.3 (-2.8 to 3.4) | -0.7 (-4.0 to 2.5) | 0.5 (-3.2 to 4.2) | 0.5 (-3.3 to 4.2) | -1.9 (-6.2 to 2.4) | -0.1 (-2.3 to 2.0) | **US-guided CSI in EPB comp** |
| ***5 (3-7)*** | ***2 (1-5)*** | ***7 (2-7)*** | ***7 (2-7)*** | ***1 (1-4)*** | ***3 (1-7)*** | ***6 (1-7)*** |

**eFigure 18.** Comparative treatment class effects expressed as coefficient with 95% CI for mid-term (13 weeks – 12 months) pain VAS. Each value represents the result of the comparison of the intervention of that column vs the intervention of that row. A negative value favours the column intervention and a positive value the row intervention. 95% CIs that do not include 0 denote statistical significance. The last row shows

© 2023 Challoumas D et al. *JAMA Network Open.*

the median rank with 95% confidence intervals for the intervention of that column. *CSI, conventional corticosteroid injection; EPB, extensor pollicis brevis; US, ultrasound*

| | | | | | | |
|---|---|---|---|---|---|---|
| **Conventional CSI** | | | | | | |
| 10.4 (6.4 to 14.4) | **Conventional CSI+thumb spica** | | | | | |
| -15.3 (-22.0 to -8.6) | -25.7 (-33.5 to -17.9) | **Thumb spica splint** | | | | |
| -3.1 (-14.6 to 8.4) | -13.5 (-25.7 to -1.4) | 12.2 (2.9 to 21.5) | **ESWT+thumb spica splint** | | | |
| -10.9 (-17.2 to -4.6) | -21.3 (-26.2 to -16.4) | 4.4 (-4.8 to 13.6) | -7.8 (-20.9 to 5.3) | **Thumb spica cast** | | |
| -13.3 (-23.5 to -3.1) | -23.7 (-34.6 to -7.8) | 2 (-5.6 to 9.6) | -10.2 (-22.3 to 1.9) | -2.4 (-14.4 to 9.5) | **As-decided thumb spica splint wear** | |
| -0.5 (-9.3 to -8.3) | -10.9 (-20.6 to -1.3) | 14.8 (3.8 to 25.8) | 2.6 (-11.9 to 17.1) | 10.3 (-0.4 to 21.2) | 12.8 (-0.6 to 26.2) | **Acupuncture** |
| ***3 (2-4)*** | ***1 (1-1)*** | ***7 (6-7)*** | ***4 (2-5)*** | ***5 (4-7)*** | ***6 (4-7)*** | ***2 (2-4)*** |

**eFigure 19.** Comparative treatment class effects expressed as coefficient with 95% CI for short-term (0-12 weeks) function (qDASH). Each value represents the result of the comparison of the intervention of that column vs the intervention of that row. A negative value favours the column intervention and a positive value the row intervention. 95% CIs that do not include 0 denote statistical significance. The last row shows the median rank with 95% confidence intervals for the intervention of that column.
*CSI, conventional corticosteroid injection; ESWT, extracorporeal shockwave therapy*

| | | |
|---|---|---|
| **Conventional CSI** | | |
| **9.4 (7,11.9)** | **Conventional CSI+thumb spica** | |
| -2.2 (-8.8 to 4.4) | -11.6 (-18.6 to -4.6) | **Thumb spica splint** |
| ***2 (2-3)*** | ***1 (1-1)*** | ***3 (2-3)*** |

**eFigure 20.** Comparative treatment class effects expressed as coefficient with 95% CI for mid-term (13 weeks – 12 months)function (qDASH). Each value represents the result of the comparison of the intervention of that column vs the intervention of that row. A negative value favours the

© 2023 Challoumas D et al. *JAMA Network Open.*

column intervention and a positive value the row intervention. 95% CIs that do not include 0 denote statistical significance. The last row shows the median rank with 95% confidence intervals for the intervention of that column

© 2023 Challoumas D et al. *JAMA Network Open.*

**Supplementary Tables**

| Study | Population (size, mean age, %F) | Duration of symptoms | Diagnostic criteria | Interventions | Duration of treatment | Follow up time points | Outcome measures | Pain score | Function score |
|---|---|---|---|---|---|---|---|---|---|
| **Peters-Velathumaningal et al,[20] 2009[N]** | **N=21** (52y, 62%) | Mean 6.5w | Pain or tenderness at the radial styloid combined with either a positive Finkelstein's test or crepitations on palpation at the radial styloid | **A)** CSI (n=12) **B)** Placebo injection (normal saline) (n=9) | 1 or 2 injections | 1w | Direct treatment response, pain severity, functional status, patient-reported improvement, adverse events | VAS | DUTCH AIMS 2-HFF |
| **Mehdinasab et al,[21] 2010[PN]** | **N=73** (33y, 86%) | Mean 6w (2-14w) | Pain at radial wrist with resisted extension or abduction of thumb; tenderness at first dorsal extensor compartment over radial styloid; positive Finkelstein's test | **A)** CSI + thumb spica cast (n=37) **B)** Thumb spica cast (n=36) | Single injection, 1m of thumb spica casting | 1m, 2m, 3m, 4m, 5m, 6m | Treatment success (no wrist pain, tenderness and Finkelstein's test) | VAS | - |

© 2023 Challoumas D et al. *JAMA Network Open.*

| Study | Population (size, mean age, %F) | Duration of symptoms | Diagnostic criteria | Interventions | Duration of treatment | Follow up time points | Outcome measures | Pain score | Function score |
|---|---|---|---|---|---|---|---|---|---|
| **Pagonis et al,[22] 2011[N]** | **N=48** (43y, 0%) | Not stated | Reporting of localised pain at dorsolateral aspect of involved wrist. Also: Pain at radial side of wrist; tenderness at first dorsal wrist extensor compartment; Positive Finkelstein's test | **A)** CSI using a 4-point injection technique (n =24) **B)** CSI using a 2-point injection technique (n=24) | Injection using either technique, with repeated injection based on success and DASH score. | 2w, 4w, 8w, 52w | **Success -** need for repeated injections, quicker successful dismissal from treatment, effect on daily activities, DASH score, relapse, side effects, and the lack or presence of surgical decompression after failure of nonsurgical treatment | - | DASH |
| **Abrisham et al,[23] 2011[P]** | **N=120** (45y, 80%) | Not stated | Positive Finkelstein's test | **A)** Surgery - Longitudinal incision (n=60) B) Surgery **-** Transverse (n=60) | Surgical procedure using either a longitudinal or transverse incision for release of first dorsal compartment | 3m | Prevalence of complications (neurovascular damage and scarring) at 3 months post surgery | - | - |

| Study | Population (size, mean age, %F) | Duration of symptoms | Diagnostic criteria | Interventions | Duration of treatment | Follow up time points | Outcome measures | Pain score | Function score |
|---|---|---|---|---|---|---|---|---|---|
| **Jongprasitkul et al,[24] 2011** | **N=40** (51y, 92.5%) | Mean 3.5w | Positive Finkelstein's test | **A)** Elastic bandaging wrapped around wrist twice, binding the base of the thumb (n=20) **B)** Neoprene thumb stabiliser (n=20) | Both groups used splint/bandage for at least 6h per day for 2 weeks. | 2w | Pain, lateral pinch strength and palmar pinch strength | VAS | - |
| **Kume et al,[25] 2012[PN]** | **N=44** (45y, 88.6%) | Mean 4.1m | History of pain in the first dorsal compartment of the wrist aggravated by excessive use and a positive Finkelstein's test | **A)** US-guided CSI (n=22) **B)** Conventional CSI (n=22) | Single injection | 4w, 6w | Pain, Proportion of patients that switched to surgery within 6 weeks after injection in each group | VAS | - |

© 2023 Challoumas D et al. *JAMA Network Open.*

| Study | Population (size, mean age, %F) | Duration of symptoms | Diagnostic criteria | Interventions | Duration of treatment | Follow up time points | Outcome measures | Pain score | Function score |
|---|---|---|---|---|---|---|---|---|---|
| **Kang et al,[26] 2013** | **N=52** (51y, 71.2%) | Mean 10.5m | Tenderness over the radial styloid; pain over first extensor compartment on resisted abduction or extension of thumb; positive Finkelstein's test | **A)** Endoscopic release of extensor compartment (n=27) **B)** Open release of extensor compartment (n=25) | Single surgical release | 2w, 6w, 12w, 24w | Pain, function, return to work time, superficial radial nerve injury, satisfaction score for scar | VAS | DASH |
| **Homayouni et al,[27] 2014** | **N=60** (46y, 55%) | >4w | Pain, swelling, tenderness over the first extensor compartment and a positive Finkelstein's test | **A)** PT – physio, paraffin bath, US under water, TENS, friction massage (n=30) **B)** KT – three type 1 pieces of kinesiology tape (n=30) | 10 sessions of PT, 4w of KT | 1m | Pain, swelling | VAS | - |

© 2023 Challoumas D et al. *JAMA Network Open.*

| Study | Population (size, mean age, %F) | Duration of symptoms | Diagnostic criteria | Interventions | Duration of treatment | Follow up time points | Outcome measures | Pain score | Function score |
|---|---|---|---|---|---|---|---|---|---|
| **Hadianfard et al,[28] 2014[N]** | **N=30** (41y, 80%) | Mean 5w | Pain and/or swelling around the styloid process of the radius and positive Finkelstein's test | **A)** Acupuncture (n=15) **B)** CSI (n=15) | A) 1 session B) 1 injection | 2w, 6w | Function, Pain | VAS | Q-DASH |
| **Mardani-Kivi et al,[29] 2014[PN]** | **N=67** (44y, 70.1%) | Not stated | Pain on radial side of wrist, tenderness at first dorsal compartment, positive Finkelstein's test and a pain score of >6 | **A)** CSI + thumb spica cast (n=33) **B)** CSI (N=34) | Both groups received a single injection<br><br>Three weeks in thumb spica cast. | 3w, 6m | Treatment success, function, pain | VAS | Q-DASH |
| **Kumar et al,[30] 2016[P]** | **N=48** (37y, 87.5%) | Mean 3.2m | Positive Finkelstein's test and no response to non-surgical treatment (diclofenac, thumb spica splint, steroid | **A)** Surgery - Longitidunal incision (n=24) **B)** Surgery - Transverse incision (n=24) | Single procedure | 6w, 3m, 6m | Pain, hypertrophic scar appearance, paraesthesia and numbness | VAS | - |

© 2023 Challoumas D et al. *JAMA Network Open.*

| Study | Population (size, mean age, %F) | Duration of symptoms | Diagnostic criteria | Interventions | Duration of treatment | Follow up time points | Outcome measures | Pain score | Function score |
|---|---|---|---|---|---|---|---|---|---|
| | | | injection) for 6 | | | | | | |
| **Orlandi et al,[31] 2015[N]** | **N=75** (45y, 68%) | Not stated | Positive Finkelstein's test | **A)** CSI (n=25)<br>**B)** CSI + 15-day delayed n. saline injection (n=25)<br>**C)** CSI + 15-day delayed hyaluronic acid injection (n=25) | A) At baseline and repeated after 15 days<br>B) At baseline and repeated after 15 days<br>C) At baseline and repeated after 15 days | 1m, 3m, 6m | Pain, function, retinaculum thickness | VAS | Q-DASH |
| **Tabinda et al,[32] 2015** | **N=50** (29y, 100%) | Not stated | Not stated | **A)** Ketoprofen phonophoresis (n=25)<br>**B)** Placebo phonophoresis (n=25) | 3 sessions per week for 4 weeks | Post-intervention | Pain perception intensity**,** Grip strength, Tip pinch, Key pinch, Palmar pinch | VAS (0-4) | None |

© 2023 Challoumas D et al. *JAMA Network Open.*

| Study | Population (size, mean age, %F) | Duration of symptoms | Diagnostic criteria | Interventions | Duration of treatment | Follow up time points | Outcome measures | Pain score | Function score |
|---|---|---|---|---|---|---|---|---|---|
| **Menendez et al,[33] 2015[N]** | **N=83** (50y, 85%) | Not stated | Based on discrete tenderness of the first extensor compartment and a positive Finkelstein's test | **A)** Full-time splint wear (n=43) **B)** As-desired splint wear (n=40) | 8w | 8w | Function**,** Grip strength, Pain intensity, Treatment satisfaction, Psychological factors on disability | NRS (VAS) | DASH |
| **Sharma et al,[34] 2015** | **N=30** (36y, 93%) | Mean 2.7m | Positive Finkelstein's test | **A)** Ultrasound therapy (n=15) **B)** Low-level laser therapy (n=15) | 7 exposures on alternate days | Post-intervention | Pain, grip strength | VAS | - |
| **Awan et al,[35] 2017** | **N=30** (31-40y, 60%) | >6m | Positive Finkelstein's test | **A)** Therapeutic ultrasound (n=15) **B)** Therapeutic ultrasound and thumb spica splint (n=15) | 12 sessions of 40 min each | Post-intervention | Function | - | Q-DASH |

© 2023 Challoumas D et al. *JAMA Network Open.*

| Study | Population (size, mean age, %F) | Duration of symptoms | Diagnostic criteria | Interventions | Duration of treatment | Follow up time points | Outcome measures | Pain score | Function score |
|---|---|---|---|---|---|---|---|---|---|
| **Lu et al,[36] 2017** | **N=51** (48y, 73%) | Not stated | Pain and tenderness located at the first dorsal compartment. | **A)** Surgery plus PRP (n=25) **B)** Surgery only (n=26) | 1 surgery +/- PRP given once | 3m, 6m, 12m | Pain, Function | PRWE | PRWE |
| **Abdulkader et al,[37] 2019** | **N=31** (?y, 71%) | Not stated | Not stated | **A)** Myofascial taping (MFT) with conventional occupational therapy n=16 **B)** Myofascial release (MFR) with conventional occupational therapy n=15 | 5w (twice a week) | 3w, 5w | Pain, Activity limitaion/functional outcome (PSFS) | VAS | PSFS (0-10) |
| **Kim et al,[38] 2019** | **N=43** (47y, 92.5%) | >3m | Not stated | **1A)** Midline incision + thumb spica splint (n=10) **1B)** Midline incision + early mobilisation | Single procedure Splint for 2 weeks | 12w, 24w | Pain, Function, Grip strength, Pinch strength | VAS | DASH |

© 2023 Challoumas D et al. *JAMA Network Open.*

| Study | Population (size, mean age, %F) | Duration of symptoms | Diagnostic criteria | Interventions | Duration of treatment | Follow up time points | Outcome measures | Pain score | Function score |
|---|---|---|---|---|---|---|---|---|---|
| | | | | (n=11)<br>**2A)** Dorsoulnar incision + thumb spica splint (n=11)<br>**2B)** Dorsoulnar incision + early mobilisation (n=11) | | | | | |
| **Ippolito et al,[39] 2020[PN]** | **N=20** (46y, ?%) | Not stated | All of: radial-sided wrist pain, first dorsal compartment tenderness, positive Finkelstein's test, and pain VAS score >3 | **A)** CSI (n=9)<br>**B)** CSI + thumb spica immobilisation (cast or splint) (n=11) | single injection, immobilisation for 3 weeks | 3w, 6m | Resolution of pain, resolution of tenderness, negative Finkelstein, pain, functional outcome | VAS | Q-DASH |

| Study | Population (size, mean age, %F) | Duration of symptoms | Diagnostic criteria | Interventions | Duration of treatment | Follow up time points | Outcome measures | Pain score | Function score |
|---|---|---|---|---|---|---|---|---|---|
| **Akhtar et al,[40] 2020[N]** | **N=134** (41y, 72%) | Not stated | Radial wrist pain, first dorsal compartment tenderness, positive Finkelstein's test | **A)** CSI +thumb cast (n=67) **B)** Thumb spica cast (n=67) | Single injection, unclear duration of immobilisation | 2w, 4w, 6w | Pain, absence of radial tenderness, negative Finkelstein, continuous pain, skin pigmentation, tenderness test positive. | VAS | Q-DASH |
| **Kumar et al,[41] 2020[PN]** | **N=120** (45y, 57%) | Not stated | Radial wrist pain, first dorsal compartment tenderness, positive Finkelstein's test and pain score >6 | **A)** CSI +thumb cast (n=60) **B)** CSI (n=60) | Single injection, cast for 4 weeks | 4w, 8w, 6m | Pain, function | VAS | Q-DASH |
| **Shin et al,[42] 2020[PN]** | **N=48** (54y, 84.1%) | Not stated | Radial wrist pain, first dorsal compartment tenderness, positive Finkelstein's test | **A)** US-guided CSI (n=24) **B)** Conventional CSI (n=24) | Single injection | 4w, 3m | Pain, function, skin pigmentation | VAS | PRWE |

© 2023 Challoumas D et al. *JAMA Network Open.*

| Study | Population (size, mean age, %F) | Duration of symptoms | Diagnostic criteria | Interventions | Duration of treatment | Follow up time points | Outcome measures | Pain score | Function score |
|---|---|---|---|---|---|---|---|---|---|
| **Haghighat et al,[43] 2021[N]** | **N=26** (46y, 62%) | Not stated | Radial tenderness, positive Finkelstein's test | **A)** ESWT + thumb spica splint (n=13) **B)** Thumb spica splint (n=13) | Three sessions over 3 weeks, Oral anti-inflammatory once daily and splint for two weeks. | 3w, 6w | Function, pain, Hand grip strength test | VAS | DASH |
| **Karlibel et al,[44] 2021** | **N=51** (50y, 88%) | Mean 6w | Positive Finkelstein's test | **A)** Paraffin bath, thumb spica splint, home exercises (n=26) **B)** Thumb spica and home exercises only (n=25) | Paraffin bath 10 sessions, over 2 weeks, thumb spica splint for 4 weeks, exercises 3 for 8 weeks | 2w, 8w | Pain, Pain pressure threshold, Handgrip strength, finger pinch strength, function, Quality of life (short form 12) | VAS | Q-DASH |
| **Das et al,[45] 2021[N]** | **N=60** (30-50y, 85%) | <6w | Not stated | **A)** Thumb spica splint (n=30) **B)** CSI (n=30) | Splint for 1 month, single injection | 1m, 3m, 6m | Pain, function | VAS | Q-DASH |

© 2023 Challoumas D et al. *JAMA Network Open.*

| Study | Population (size, mean age, %F) | Duration of symptoms | Diagnostic criteria | Interventions | Duration of treatment | Follow up time points | Outcome measures | Pain score | Function score |
|---|---|---|---|---|---|---|---|---|---|
| **Başar et al,[46] 2021[PN]** | **N=84** (48y, 73%) | Not stated | Radial wrist pain, first dorsal compartment tenderness, positive Finkelstein's test | **A)** CSI (n=42) **B)** CSI + thumb spica splint (n=42) | Sigle injection, splint for 1 month | 12m | Function, Pain, Finkelstein's test. | VAS | Q-DASH |
| **Senlikci et al,[47] 2021[N]** | **N=36** (48y, 83%) | Mean 6m | Positive Finkelstein's test | **A)** Neural therapy, thumb spica splint, rest (n=18) **B)** Thumb spica splint, rest (n=18) | Neural therapy for 2 weeks, duration of thumb spica and rest unclear | 1m, 12m | Pain, function, Finkelstein's test. | VAS | Duruöz hand index (0-90) |
| **Salim et al,[48] 2021** | **N=40** (42y, 75%) | Mean 8.1m, all >6m | "Clinical diagnosis of de Quervain's" | **A)** Surgery – pulley release (n=20) **B)** Surgery – pulley reconstruction (n=20) | Single procedure | 1w, 3m, 6m | Pain, function, tendon subluxation | VAS | Q-DASH, Mayo wrist score |

© 2023 Challoumas D et al. *JAMA Network Open.*

| Study | Population (size, mean age, %F) | Duration of symptoms | Diagnostic criteria | Interventions | Duration of treatment | Follow up time points | Outcome measures | Pain score | Function score |
|---|---|---|---|---|---|---|---|---|---|
| **Jung et al,[49] 2022[N]** | **N=48** (56y, 92%) | Mean 3m | History, examination, confirmed with US | **A)** US-guided CSI in both compartments (n=24) **B)** US-guided steroid injection in EPB compartment only (n=24) | Single injection | 6w, 3m | Pain, Complications – hypopigmentation, proceeding to surgery. | VAS | - |

**Supplementary Table 1. Characteristics of the included randomised controlled trials.**
[P]: Participated in pairwise meta-analyses.
[N]: participated in network meta-analyses.
*APL, abductor pollicis longus; APB, abductor pollicis brevis; CSI, corticosteroid injection; DASH, disabilities of the arm, shoulder and hand; EPB, extensor pollicis brevis; ESWT, extracorporeal shockwave therapy; m, months; QDASH, quick DASH; TENS, transcutaneous electrical nerve stimulation; US, ultrasound; VAS, visual analogue scale; w, weeks, y, years.*

© 2023 Challoumas D et al. *JAMA Network Open.*

| Comparison | Study | Pain VAS | | MD pain VAS (95% CI) | | Function | | | MD function (95% CI) | |
|---|---|---|---|---|---|---|---|---|---|---|
| | Follow up | ST | MT | ST | MT | ST | MT | Scale | ST | MT |
| CSI + thumb spica (1) vs CSI (2) | Mardani-Kivi et al. (2014) | ↑ | ↑ | 1.3 (0.5 to 2.1) | 1.5 (0.6 to 2.4) | ↑ | ↑ | qDASH | 10.0 (4.2 to 15.8) | 10.0 (6.0 to 14.0) |
| | Ippolito et al. (2020) | ↔ | ↔ | 0.1 (-0.9 to 1.1) | 0 (-0.8 to 0.8) | ↔ | ↔ | qDASH | 11.3 (-1.5 to 24.1) | 11.7 (0.6 to 22.8) |
| | Kumar et al. (2020) | ↑ | ↑ | 2.0 (1.6 to 2.4) | 2.5 (2.0 to 3.0) | ↑ | ↑ | qDASH | 10.7 (5.7 to 15.7) | 9.8 (5.1 to 14.5) |
| | Basar et al. (2021) | - | ↑ | - | 0.8 (0.4 to 1.1) | - | ↑ | qDASH | - | 8.0 (3.6 to 12.4) |
| **Overall CSI + thumb spica (1) vs CSI (2) (Certainty of Evidence)** | | **↑ (v. low)** | **↑ (v. low)** | **1.3 (0.4 to 2.1)** | **1.2 (0.3 to 2.1)** | **↑ (moderate)** | **↑ (moderate)** | qDASH | **10.5 (6.8 to 14.1)** | **9.42 (7.0 to 11.9)** |
| US-guided CSI (1) vs Conventional CSI (2) | Kume et al. (2012) | ↑ | - | 3.4 (2.2 to 4.6) | - | - | - | - | - | - |
| | Shin et al. (2020) | ↔ | ↔ | 0.8 (-1.8 to 0.2) | 0.4 (-0.6 to 1.4) | ↔ | ↔ | PRWE-F | -1 (-6.1 to 4.1) | -2.4 (-7.5 to 2.7) |

© 2023 Challoumas D et al. *JAMA Network Open.*

| Comparison | Study<br>Follow up | Pain VAS ST | Pain VAS MT | MD pain VAS (95% CI) ST | MD pain VAS (95% CI) MT | Function ST | Function MT | Scale | MD function (95% CI) ST | MD function (95% CI) MT |
|---|---|---|---|---|---|---|---|---|---|---|
| **Overall US-guided CSI (1) vs Conventional CSI (2) (Certainty of Evidence)** | | **↔ (v. low)** | - | **2.1 (-0.5 to 4.6)** | - | - | - | - | - | - |
| **CSI (1) vs placebo injection (n. saline) (2)** | Peters-Velathumaningal et al. (2009) | ↑ | - | 3.0 (1.3 to 4.7) | - | - | - | - | - | - |
| **CSI + thumb spica cast (1) vs thumb spica cast (2)** | Mehdinasab et al. (2010) | - | ↑ | - | 1.4 (1.1 to 1.7) | - | - | - | - | - |
| **CSI 4-point technique (1) vs CSI 2-point technique (2)** | Pagonis et al. (2011) | - | - | - | - | ↑ | ↑ | DASH | 18.8 (11.4 to 26.2) | 5.9 (1.6 to 10.2) |
| **Elastic bandage(1) vs thumb spica splint (2)** | Jongprasitkul et al. (2011) | ↑ | - | 1.3 (0.6 to 2.0) | - | - | - | - | - | - |
| **Open surgery (1) vs Endoscopic surgery (2)** | Kang et al. (2013) | ↓ | ↔ | -2 (-2.9 to -1.1) | 0 (-0.8 to 0.8) | ↔ | ↔ | DASH | -5.4 (-13.7 to 2.9) | 1.3 (-5.4 to 8.0) |
| **Physiotherapy (1) vs Kinesio-tape (2)** | Homayouni et al. (2014) | ↓ | - | -2.3 (variance data not provided) | - | - | - | - | - | - |
| **CSI (1) vs Acupuncture (2)** | Hadianfard et al. (2014) | ↔ | - | 0.5 (-0.8 to 1.8) | - | ↔ | - | qDASH | 1.5 (-7.3 to 10.3) | - |
| **CSI (1) vs CSI + delayed placebo (n. saline) injection (2)** | Orlandi et al. (2015) | ↔ | ↓ | 0 (-0.4 to 0.4) | -1.0 (-1.5 to -0.5) | ↔ | ↔ | qDASH | -1.6 (-3.7 to 1.5) | -1.3 (-3.9 to 1.3) |
| **CSI (1) vs CSI + delayed HA injection (2)** | Orlandi et al. (2015) | ↔ | ↓ | 0 (-0.5 to 0.5) | -2.0 (-2.6 to -1.4) | ↔ | ↓ | qDASH | -1.5 (-13.7 to 10.7) | -25.5 (-27.8 to -25.2) |
| **CSI + delayed HA injection (1) vs CSI + delayed placebo (n. saline) injection (2)** | Orlandi et al. (2015) | ↔ | ↑ | 0 (-0.5 to 0.5) | 1.0 (-0.3 to 1.7) | ↔ | ↓ | qDASH | 0.1 (-0.3 to 0.4) | 24.2 (21.8 to 26.6) |
| **Full-time thumb spica splint wear (1) As-** | Menendez et al. (2015) | ↔ | - | -0.1 (-1.1 to 0.9) | - | ↔ | - | - | -3 (-10.6 to 4.6) | - |

© 2023 Challoumas D et al. *JAMA Network Open.*

| Comparison | Study Follow up | Pain VAS ST | Pain VAS MT | MD pain VAS (95% CI) ST | MD pain VAS (95% CI) MT | Function ST | Function MT | Scale | MD function (95% CI) ST | MD function (95% CI) MT |
|---|---|---|---|---|---|---|---|---|---|---|
| **decided thumb spica splint wear (2)** | | | | | | | | | | |
| **US therapy (1) vs Low-level laser therapy (2)** | Sharma et al. (2015) | ↔ | - | 0.4 (-1.7 to 2.5) | - | - | - | - | - | - |
| **US therapy (1) vs US therapy + thumb spica splint (2)** | Awan et al. (2017) | ↓ | - | -0.9 (-1.3 to -0.5) | - | - | - | - | - | - |
| **Open surgery + PRP injection (1) vs open surgery (2)** | Lu et al. (2017) | ↔ | ↑ | -0.2 (-0.5 to 0.1) | 0.8 (0.5 to 1.1) | - | - | - | - | - |
| **Myofascial release + occupational therapy (1) Myofascial taping + occupational therapy (2)** | Abdulkader et al. (2019) | ↑ | - | Exact data not given | | ↑ | - | PSFS (patient-specific functional scale) | Exact data not given | - |
| **Open surgery (midline incision) (1) vs Open surgery (dorsoulnar incision) (2)** | Kim et al. (2019) | ↔ | ↔ | -0.9 (variance data not provided) | -0.2 (variance data not provided) | ↔ | ↔ | DASH | -4 (variance data not provided) | -5 (variance data not provided) |
| **Open surgery (longitudinal incision) (1) vs Open surgery (transverse incision) (2)** | Kumar et al. (2016) | - | ↔ | - | 0.3 (-1.1, 2.7) | - | - | - | - | - |
| **CSI + thumb spica cast (1) thumb spica cast (2)** | Akhtar et al. (2020) | ↑ | - | 1.8 (1.3 to 2.3) | - | ↑ | - | - | 21.3 (16.5 to 26.1) | - |
| **ESWT + thumb spica splint (1) thumb spica splint (2)** | Haghighat et al. (2021) | ↑ | - | 2.7 (1.8 to 3.6) | - | ↑ | - | - | 12.2 (2.9 to 21.5) | - |
| **Thumb spica splint + home exercises + paraffin bath (1) vs** | Karlibel et al. (2021) | ↑ | - | 1 (1.7) | - | ↑ | - | qDASH | 4.8 (14.3) | - |

© 2023 Challoumas D et al. *JAMA Network Open.*

| Comparison | Study | Pain VAS | | MD pain VAS (95% CI) | | Function | | | MD function (95% CI) | |
|---|---|---|---|---|---|---|---|---|---|---|
| | **Follow up** | **ST** | **MT** | **ST** | **MT** | **ST** | **MT** | **Scale** | **ST** | **MT** |
| **Thumb spica splint + home exercises (2)** | | | | | | | | | | |
| **CSI (1) vs thumb spica splint (2)** | Das et al. (2021) | ↑ | ↔ | 1.4 (0.8 to 2.0) | 0.2 (-0.3 to 0.7) | ↑ | ↔ | - | 15.3 (8.8 to 21.8) | 2.2 (-4.4 to 8.8) |
| **Neural therapy + thumb spica splint + rest (1) vs thumb spica splint + rest (2)** | Senlikci et al. (2021) | ↑ | ↑ | 2.8 (2.0 to 3.6) | 2.4 (1.4 to 3.4) | ↑ | ↑ | - | 21.0 (14.0 to 28.0) | 17.2 (8.1 to 26.3) |
| **Open surgery - pulley release (1) vs Open surgery - pulley reconstruction (2)** | Salim et al. (2021) | - | ↔ | - | 0.1 (-0.6 to 0.8) | - | ↔ | qDASH | - | -1.5 (-10.8 to 7.8) |
| **US-guided injection in EPB compartment only (1) vs US-guided injection in both compartments (2)** | Jung et al. (2022) | ↔ | | 0 (-0.6 to 0.6) | -0.1 (-1.0 to 0.8) | - | - | - | - | - |

**eTable 1.** Results of each study showing the mean difference between the compared interventions for pain and function. The up and down arrows represent statistical significance. Up arrow and positive value for both pain and function favours intervention 1, down arrow and negative value favours intervention 2.

*CSI, corticosteroid injection; ESWT, extracorporeal shockwave therapy; HA, hyaluronic acid; MT, mid-term; PRP, platelet-rich plasma; ST, short-term; US, ultrasound*

© 2023 Challoumas D et al. *JAMA Network Open.*

| Study – RoB 2 domain | Randomisation | Deviations from intended protocol | Missing data | Measurement of outcome | Selection of result | Overall RoB |
|---|---|---|---|---|---|---|
| Menendez et al. (2015) | High | Some concerns | High | Some concerns | Low | High |
| Orlandi et al. (2015) | Some concerns | Some concerns | Low | Some concerns | Low | Some concerns |
| Ippolito et al. (2020) | Some concerns | Some concerns | Some concerns | Low | Low | Some concerns |
| Jung et al. (2022) | Low | Some concerns | Low | Some concerns | Low | Some concerns |
| Kumar et al. (2020) | Some concerns | Some concerns | Low | Some concerns | Low | High |
| Senlikci et al. (2019) | High | Some concerns | Low | High | Low | High |
| Mardani-Kivi et al. (2014) | Some concerns | Some concerns | Low | Some concerns | Low | Some concerns |
| Hadianfard et al. (2014) | Some concerns | Some concerns | High | Some concerns | Low | Some concerns |
| Akhtar et al. (2020) | Some concerns | Some concerns | Low | Some concerns | Low | High |
| Haghighat et al. (2021) | High | Low | Low | Some concerns | Low | High |
| Jongprasitkul et al. (2011) | High | Some concerns | Low | Some concerns | Low | High |
| Kume et al. (2012) | Some concerns | High | Low | Low | Low | Some concerns |
| Peters-Velathumaningal et al. (2009) | Low | Low | Low | Low | Low | Low |
| Basar et al. (2021) | Low | Some concerns | Some concerns | Some concerns | Low | Some concerns |
| Das et al. (2021) | Low | Some concerns | Low | Some concerns | Low | Some concerns |
| Shin et al. (2020) | Low | Some concerns | Low | Low | Low | Some concerns |
| Mehdinasab et al. (2010) | Some concerns | Some concerns | Low | Some concerns | Low | Some concerns |
| Abrisham et al. (2011) | Some concerns | Some concerns | Low | Some concerns | Low | High |
| Kumar (2016) | Some concerns | Some concerns | Low | Low | Low | High |

© 2023 Challoumas D et al. *JAMA Network Open.*

**eTable 2.** Results of risk of bias assessment using the Cochrane RoB 2 tool. *RoB, risk of bias*

© 2023 Challoumas D et al. *JAMA Network Open.*

| Comparison | Outcome measure | Follow up | Number of studies | Overall risk of bias | Inconsistency | Indirectness | Imprecision | Other | Certainty of Evidence |
|---|---|---|---|---|---|---|---|---|---|
| **Conventional CSI + thumb spica vs Conventional CSI alone** | **Pain** | Short-term | 3 | High | High (-2) | Low | Low | Low | ⊕∅∅∅ V. low |
| | | Mid-term | 4 | High | High (-2) | Low | Low | Low | ⊕∅∅∅ V. low |
| | **Function** | Short-term | 3 | High | Low | Low | Low | Low | ⊕⊕⊕∅ Moderate |
| | | Mid-term | 4 | High | Low | Low | Low | Low | ⊕⊕⊕∅ Moderate |
| **Conventional CSI vs US-guided CSI** | **Pain** | Short-term | 2 | High | High (-2) | Low | Low | Low | ⊕∅∅∅ V. low |
| **Transverse vs Longitudinal skin incision** | **Total complications** | Mid-term | 2 | High | High | Low | High (-2) | Low | ⊕∅∅∅ V. low |
| | **Nerve injury** | Mid-term | 2 | High | Low | Low | High (-2) | Low | ⊕∅∅∅ V. low |
| | **Scar hypertrophy** | Mid-term | 2 | High | Low | Low | High (-2) | Low | ⊕∅∅∅ V. low |

**eTable 3.** Results of certainty of evidence assessment using the GRADE tool. For inconsistency, the certainty of evidence was downgraded by one step where statistical heterogeneity was significant ($I^2$=50-80%) and by two steps where it was substantial ($I^2$>80%). For imprecision, the certainty of evidence was downgraded by one step if the confidence interval was wide or the optimal information size was not met and by two steps where the confidence interval was very wide. The results of all comparisons were downgraded by one step for overall risk of bias and there was no downgrading for indirectness (clinical heterogeneity) or “other” as publication bias was not assessed formally (no comparisons included more than ten studies).

© 2023 Challoumas D et al. *JAMA Network Open.*

| Total studies n=11 | Largest contribution | Mean difference (95% CI) | Certainty of Evidence | Median ranking (95% CI) | Interpretation of Findings |
|---|---|---|---|---|---|
| **CSI (A)** | - | - | - | 9 (6-12) | Reference comparator |
| **CSI + thumb spica cast (B)** | Direct estimate (3 RCTs) | 0.9 (-0.5 to 2.4) | ⊕∅∅∅ V. low[B,I] | 7 (2-11) | Probably superior |
| **Thumb spica splint (C)** | Direct estimate (1 RCT) | -1.4 (-3.3 to 0.5) | ⊕⊕⊕∅ Moderate[B] | 12 (9-14) | Probably inferior |
| **US-guided CSI + HA injection (D)** | Indirect estimate | 2.1 (-1.0 to 5.1) | - | 1 (1-7) | Probably superior |
| **US-guided CSI + placebo injection (E)** | Indirect estimate | 2.1 (-1.0 to 5.1) | - | 1 (1-7) | Probably superior |
| **Neural therapy + thumb spica cast (F)** | Indirect estimate | 1.4 (-1.4 to 4.2) | - | 1 (1-10) | Probably superior |
| **ESWT + thumb spica splint (G)** | Indirect estimate | 1.3 (-1.5 to 4.1) | - | 1 (1-10) | Probably superior |
| **Thumb spica cast (H)** | Indirect estimate | 0.1 (-2.1 to 2.4) | - | 10 (5-13) | Probably superior |
| **As decided spica splint wear (I)** | Indirect estimate | -1.3 (-4.1 to 1.5) | - | 13 (8-14) | Probably inferior |
| **US-guided CSI in EPB compartment only (J)** | Direct estimate (1 RCT) | 2.1 (-1.0 to 5.2) | ⊕⊕⊕⊕ High[B*] | 1 (1-6) | Definitely superior |
| **Acupuncture (K)** | Direct estimate (1 RCT) | -0.5 (-2.7 to 1.7) | ⊕⊕⊕∅ Moderate[B] | 10 (5-14) | Probably inferior |
| **Elastic bandage (L)** | Indirect estimate | -0.1 (-2.9 to 2.7) | - | 11 (5-13) | Probably inferior |
| **US-guided CSI (in both compartments) (M)** | Indirect estimate | 2.1 (0.2 to 3.9) | - | 3 (1-6) | Probably superior |
| **Placebo injection (N)** | Direct estimate (1 RCT) | -3.0 (-6.0 to -0.1) | ⊕⊕⊕∅ Moderate[B] | 14 (11-14) | Definitely inferior |

**eTable 4.** Summary of findings table for short-term (0-12 weeks) pain VAS. In the interpretation of findings column, an intervention was “definitely” (instead of probably) superior or inferior to the reference intervention when both statistical and clinical significance were reached and the certainty of evidence was at least moderate.

[B]: Downgraded due to overall risk of bias

[I]: downgraded due to inconsistency

*: upgraded due to large magnitude of effect

*CSI, conventional corticosteroid injection; EPB, extensor pollicis brevis; ESWT, extracorporeal shockwave therapy; HA, hyaluronic acid; RCT, randomised clinical trial; US, ultrasound*

© 2023 Challoumas D et al. *JAMA Network Open.*

| Total studies n=7 | Largest contribution | Mean difference (95% CI) | Certainty of Evidence | Median ranking (95% CI) | Interpretation of Findings |
|---|---|---|---|---|---|
| **CSI (A)** | - | - | - | 5 (3-7) | Reference comparator |
| **CSI + thumb spica cast (B)** | Direct estimate (4 RCTs) | 1.0 (0.1 to 2.1) | ⊕∅∅∅ V. low[B,I] | 2 (1-5) | Probably superior |
| **Thumb spica splint (C)** | Direct estimate (1 RCT) | -0.2 (-2.2 to 1.8) | ⊕⊕⊕∅ Moderate[B] | 7 (2-7) | Probably inferior |
| **Thumb spica cast (D)** | Indirect estimate | -0.2 (-2.4 to 2.1) | - | 7 (2-7) | Probably inferior |
| **Neural therapy + thumb spica cast (E)** | Indirect estimate | 2.2 (-0.8 to 5.2) | - | 1 (1-4) | Probably superior |
| **US-guided CSI** | Direct estimate (1 RCT) | 0.4 (-1.8 to 2.6) | ⊕∅∅∅ V. low[B,I] | 3 (1-7) | Probably superior |
| **US-guided CSI in EPB comp only** | Indirect estimate | 0.3 (-2.8 to 3.4) | - | 7 (1-7) | Probably superior |

**eTable 5.** Summary of findings table for mid-term (13 weeks – 12 months) pain VAS. In the interpretation of findings column, an intervention was “definitely” (instead of probably) superior or inferior to the reference intervention when both statistical and clinical significance were reached and the certainty of evidence was at least moderate

[B]: Downgraded due to overall risk of bias

[I]: downgraded due to inconsistency

*CSI, conventional corticosteroid injection; EPB, extensor pollicis brevis; RCT, randomised clinical trial; US, ultrasound*

© 2023 Challoumas D et al. *JAMA Network Open.*

| Total studies n=8 | Largest contribution | Mean difference (95% CI) | Certainty of Evidence | Median ranking (95% CI) | Interpretation of Findings |
|---|---|---|---|---|---|
| **CSI (A)** | - | - | - | 3 (2-4) | Reference comparator |
| **CSI + thumb spica cast (B)** | Direct estimate (3 RCTs) | 10.5 (6.8 to 14.1) | ⊕⊕⊕∅ Moderate[B] | 1 (1-1) | Probably superior |
| **Thumb spica splint (C)** | Direct estimate (1 RCT) | -15.3 (-22.0 to -8.6) | ⊕⊕⊕⊕ High[B,*] | 7 (6-7) | Definitely inferior |
| **ESWT + thumb spica splint (D)** | Indirect estimate | -3.1 (-14.6 to 8.4) | - | 4 (2-5) | Probably inferior |
| **Thumb spica cast (E)** | Indirect estimate | -10.9 (-17.2 to -4.6) | - | 5 (4-7) | Probably inferior |
| **As decided spica splint wear (F)** | Indirect estimate | -13.3 (-23.5 to -3.1) | - | 6 (4-7) | Probably inferior |
| **Acupuncture (G)** | Direct estimate (1 RCT) | -0.5 (-9.3 to -8.3) | ⊕⊕⊕∅ Moderate[B] | 2 (2-4) | Probably inferior |

**eTable 6.** Summary of findings table for short-term (0-12 weeks) function qDASH. In the interpretation of findings column, an intervention was "definitely" (instead of probably) superior or inferior to the reference intervention when both statistical and clinical significance were reached and the certainty of evidence was at least moderate.
[B]: Downgraded due to overall risk of bias
*: upgraded due to large magnitude of effect
*CSI, conventional corticosteroid injection; ESWT, extracorporeal shockwave therapy; RCT, randomised clinical trial.*

| Total studies n=5 | Largest contribution | Mean difference (95% CI) | Certainty of Evidence | Median ranking (95% CI) | Interpretation of Findings |
|---|---|---|---|---|---|
| **CSI (A)** | - | - | - | 2 (2-3) | Reference comparator |
| **CSI + thumb spica cast (B)** | Direct estimate (4 RCTs) | 9.4 (7.0 to 11.9) | ⊕⊕⊕∅ Moderate[B] | 1 (1-1) | Probably superior |
| **Thumb spica splint (C)** | Direct estimate (1 RCT) | -2.2 (-8.8 to 4.4) | ⊕⊕⊕∅ Moderate[B] | 3 (2-3) | Probably inferior |

**eTable 7.** Summary of findings table for mid-term (13 weeks – 12 months) function qDASH. In the interpretation of findings column, an intervention was "definitely" (instead of probably) superior or inferior to the reference intervention when both statistical and clinical significance were reached and the certainty of evidence was at least moderate.
[B]: Downgraded due to overall risk of bias
*CSI, conventional corticosteroid injection; RCT, randomised clinical trial*

© 2023 Challoumas D et al. *JAMA Network Open.*
