# Supplementary material for: Management of de Quervain Tenosynovitis: A Systematic Review and Network Meta-Analysis
Source: JAMA Netw Open. 2023 Oct 27;6(10):e2337001. doi: 10.1001/jamanetworkopen.2023.37001 (PMC10611995; doi:10.1001/jamanetworkopen.2023.37001)
Supplement: Supplement 2. — Data Sharing Statement [file jamanetwopen-e2337001-s002.pdf]

Data Sharing Statement

Challoumas. Management of de Quervain Tenosynovitis. *JAMA Netw Open*. Published October 20, 2023. doi:10.1001/jamanetworkopen.2023.37001

Data
Data available: Yes
Data types: Other (please specify)
Additional Information: All the analysis and data will be made available from the First Author (DC)
How to access data: dchalloumas@hotmail.co.uk
When available: With publication

Supporting Documents
Document types: None

Additional Information
Who can access the data: Anyone requesting the data
Types of analyses: For any purpose or for a specified purpose
Mechanisms of data availability: After approval of a proposal
